# Fourier analysis of signal dependent noise images

**DOI:** 10.1038/s41598-024-78299-1

**Published:** 2024-12-28

**Authors:** John Heine, Erin Fowler, Matthew B. Schabath

**Affiliations:** Cancer Epidemiology Department, H. Lee Moffitt Cancer Center and Research Institute, 12902 Bruce B. Downs Blvd, Tampa, FL 33612 USA

**Keywords:** Signal dependent noise, Mammography, Mammographic simulations, Wavelet expansion, Fourier analysis, Bioinformatics, Optical imaging, Image processing, Optical physics, X-rays

## Abstract

An archetype signal dependent noise (SDN) model is a component used in analyzing images or signals acquired from different technologies. This model-component may share properties with stationary normal white noise (WN). Measurements from WN images were used as standards for making comparisons with SDN in both the image domain (ID) and Fourier domain (FD). The ID wavelet expansion was applied to WN images (n = 1000). Orthogonality conditions were used to parametrically model the variance decomposition, as described in both domains. FD components were investigated with probability density function modeling and summarized measures. SDN images were constructed by multiplying both *simulated* and clinical mammograms (both with n = 1000) by WN. The variance decomposition for both WN and SDN decreases exponentially as a parametric function of the ID expansion level; expansion image variances for both types of noise were captured similarly in the Fourier plane corresponding with the ID parametric model. The Fourier transform of WN has a uniform power spectrum distributed exponentially; SDN has similar attributes. Fourier inversion of the lag-autocorrelation performed in the FD produced a statistical estimation of the SDN’s image factor. These findings are counterintuitive as SDN can be nonstationary in the ID but have stationary attributes in the FD.

## Introduction

### Signal dependent noise prevalence

In many situations, noise variation depends on the signal^1–4^, commonly referenced as signal dependent noise (SDN). For example, this noise is present in the following mechanisms: digitized optical film^2,5,6^; magnetic tape-recordings^7,8^; in images taken with digital cameras^9–12^, synthetic aperture radar (SAR)^2,13–21^, ultrasound (US)^2,16,19,20,22–25^, and mammography^26–30^; and video^31^. In general, noise variation can be either a linear or non-linear function of the pixel’s expectation^4^. One source of SDN occurs when the detection is based on photon flux interaction (*light measurements*) as with complementary metal oxide semiconductors (CMOSs) and charge-coupled devices (CCDs)^9,11,29^. These semiconductors operate by the photoelectric effect^32,33^ and are used in many digital imaging applications^33,34^; SDN is inherent in such systems and is referred to as shot noise (i.e., synonymous with photon noise or Poisson noise). Similarly, standard two-dimensional (2D) mammography images are also affected by shot noise as a consequence of measuring a Poisson process^35^. CCD and CMOS systems or standard mammography operate by incoherent detection because the captured signal is not dependent upon its phase. Speckle noise is also signal dependent and present in coherent imaging systems (i.e., phase of incoming signal is important), such as US and SAR, where it is caused by destructive and constructive interference^15,19,36^.

### Signal dependent noise modeling

This section reviews a general model for images corrupted with SDN with specific examples to put the focus of this current study in context. The SDN model-type studied here is a component used in modeling images captured with varied devices^2,9^ and is expressed as

1 $${{\rm{I}}_{{\rm{c}}}} ( {{\rm{x, y}}} ) = {{\rm{c}}_{1}} \times {\rm{I(x, y)}} + {{\rm{c}}_{2}} \times {\rm{I(x, y)}}^{{\upalpha }} \times {\rm{u(x, y)}}.$$

Here, I_c_(x, y) is the noise corrupted image, I(x, y) is the noiseless image, the exponent, ɑ, is often assumed valid over [0,1], u(x, y) is zero mean random noise in this form, the two scaling constants are greater than or equal to zero, I(x, y)^α^ × u(x, y) is the multiplicative SDN term, and (x, y) are pixel coordinates^9,11,13,16,20,37^. An independent additive random noise term is often included to account for thermal or electronic noise, and the scaling constants are frequently set to unity, or equivalently incorporated into the other terms. With adjustments, this model has been applied to SAR, US^14,16,20–22,24,25,37–39^, and mammography^26^. More broadly, the model is applicable to systems that use either CCD^9^ or CMOS semiconductors^29,40^. An approximation for shot noise is given by

2 $${\text{I}}_{\text{c}}\left(\text{x},\text{ y}\right)={\text{c}}_{1}\times \text{I}\left(\text{x},\text{ y}\right)+ \sqrt{{\text{c}}_{1}}\times \text{I}(\text{x},\text{ y}{)}^\frac{1}{2}\times \text{u}\left(\text{x},\text{ y}\right),$$

where u(x, y) is zero-mean normal. This form is analogous letting I_c_(x,y) be distributed normally with mean = c_1_ × I(x, y) and variance = c_1_ × I(x, y) [or more generalized by letting mean ~ variance]. Examples of using the Eq. (2) model in the analyses of CMOS and CCD images are provided by various articles^4,29,40,41^.The speckle model for SAR images can be expressed as

3 $${\text{I}}_{\text{c}}\left(\text{x},\text{ y}\right)=\text{I}\left(\text{x},\text{ y}\right)\times \text{u}\left(\text{x},\text{ y}\right),$$

where u(x, y) is random noise with non-zero positive mean^36^. The random noise factor, u(x, y), is often assumed to follow a Gamma distribution in the SAR model with parameters dependent upon the acquisition technique^14,21,24,37^. The US medical image model corrupted with speckle noise^20,22,38^ can be framed similar to the SAR model but with u(x, y) assumed normally^22^ or uniformly distributed^38^.

Research investigating SDN in both coherent and non-coherent imaging systems spans many years. We have categorized this work broadly in two related problems of either (i) analyzing signal and noise relationships, and (ii) noise suppression algorithm developments, where the first task is often used for the second task. Early work in film-gran analysis was concerned with deriving optimal and sub-optimal mean square error estimations^8,42^. Mean square estimators were derived for video by using inter-frame redundancy with low–high frequency analyses for noise suppression purposes^29^. Other work focused on determining model parameters and signal-variance relationships for noise reduction in CCD camera images^4^, characterizing noise in images taken with CMOS or CCD sensors by examining the entire camera systems^9,40^, and estimating noise in CMOS images using Fisher information^40^. Another approach used the Anscombe transformation to convert CCD images (i.e., a shot noise transformation) into a form approximating signal with additive normal noise before denoising^10^. The Anscombe transformation was also used in mammography: (i) for synthesizing SDN images to investigate dose reductions^26^; and (ii) for both adaptive noise suppression and image enhancement^43^. Related work estimated the model parameters to apply adaptive optimized mean square filtering evaluated with synthetic SDN images^2^. An earlier review of noise reduction techniques for SAR images was given by Lee* et al*.^17^. Work by Kuan* et al*. developed an adaptive smoothing technique based on the specific type of SDN model with film-grain and Poisson noise as examples^44^. Schulze and Wu introduced nonlinear filtering that maintains edge detail in SAR images^18,21^. Other work investigated locally adaptive filtering of SAR images by first mapping the data into a signal plus noise form^16^, similar to the objective of the Anscombe work. Similarly, adaptive filtering using fuzzy logic was used to remove noise while maintaining edges in SAR images^13^. Other work developed methods to investigate speckle noise in SAR images to understand its effects^36,45^. Anisotropic diffusion filtering followed by wavelet noise suppression was applied US images^39^. Similar to the Anscombe approach, several filtering techniques were applied to medical US images after transforming the data into a form resembling signal with additive normal noise^24^, whereas Khan* et al*.^22^ applied denoising with the Schur decomposition. Reviews of speckle noise reduction techniques are provided elsewhere^19,20^, and a survey of statistical modeling techniques of SAR images by Gao^14^.

### Study motivation

Similarities between white noise (WN) and SDN were observed in prior studies of mammographic breast density research^46,47^ but were not investigated in detail at that time. Mammographic breast density (or breast density) is a clinically and statistically significant breast cancer risk factor with important translational implications^48^. Our understanding of SDN is an evolving process dependent upon the SDN functional relationship with the signal^27,28,46,49^. Ultimately, this understanding is fundamental to our automated breast density detection algorithm’s performance. In this investigation, both simulated and clinical mammograms were used to create SDN with a multiplicative model (i.e., image × noise). Here, we used real mammograms as approximations for clean or noiseless images. Stochastic images with a $$1\!\left/ \raisebox{-0.2ex}{${\text{f}}^{2\upbeta }$}\right.$$ power spectra were used as *simulated* mammograms by adjusting $$\upbeta$$ accordingly^49–55^, noting these images can be considered as fractal^56^ for the value of $$\upbeta$$ = 1.5 used in this investigation.

This present investigation’s focus is the multiplicative SDN component of Eq. (3) in isolation. As a generalization, we first note that the clean image can be expressed as a general memoryless function^42^ of another noiseless image given by

4 $$\text{I}\left(\text{x},\text{ y}\right)=\text{z}\left(\text{q}\left(\text{x},\text{ y}\right)\right),$$

where z(∙) is some general function, and q(x, y) is another related image without noise. The SDN component I(x, y) × u(x, y) with zero mean u(x, y)^42^ is the central point of this present study; noting, this constituent is either explicitly (i.e., shot noise model) or implicitly (i.e., SAR and US models) the SDN component in these image models discussed above (i.e., Eqs. 1–3). There is no loss of generality in this study when setting α = 1 as will become clear below.

### Objectives

We applied multiple strategies for comparing normally distributed stationary WN with SDN. Known or predicable characteristics of WN were established as metrics. These metrics were then used as comparators for SDN undergoing the same analyses. Comparisons were made in both the image domain (ID) and Fourier domain (FD). The ID wavelet expansion was used as a modeling simplification mechanism. Wavelet techniques have been used in countless applications, as addressed in published reviews^57–62^ covering both background and advances. The theoretical foundation for the wavelet forward transform and inversion can be presented on many levels of complexity. We have chosen to use an intuitive ID expansion approach not steeped in theory. A portion of the study was also performed in the FD, aside from the wavelet analyses. Study objectives are organized about four analytical strategies applied sequentially to WN to develop standards for comparison as summarized:

- Strategy 1: the ID wavelet expansion’s orthogonality conditions were used to decompose the image variance.
- Strategy 2: the decomposed variance was examined in the FD and its spatial distribution in the 2D frequency plane was illustrated with conceptual graphical layouts.
- Strategy 3: the ID variance decomposition was investigated with parametric modeling and the related spectral properties were compared with the illustration in Strategy 2.
- Strategy 4: images were characterized in the FD without the wavelet transform.

## Materials and methods

### Background, study overview, and conventions

Stationary random processes are often discussed with one-dimensional (1D) stochastic signals measured over time. In such processes (stationary in the strict sense), the ensemble’s statistical properties are invariant with respect to translations in time. Here, we use a time invariant 1D ensemble example from Thomas^63^ and then generalize to a 2D image system. This ensemble is comprised of many same-type resistors held at the same temperature. The thermal voltage across each resistor is then measured as a function of time. Under these conditions, ensemble statistical properties would not be time dependent because the physical basis for each signal is static in time. Extending this example to an ensemble comprised of images, a stationary 2D random process would have statistical properties invariant with respect to spatial translations. As a general example, this implies that many images of the same 2D spatially stationary phenomena were acquired repeatedly to form the ensemble. Then, statistical properties would not be a function of spatial coordinates. We can consider ID and FD as two separate but related processes. Although not intuitive, the Fourier transform (FT) of a random process creates another random process that has its own characteristics^63^.

**Fig. 1:**
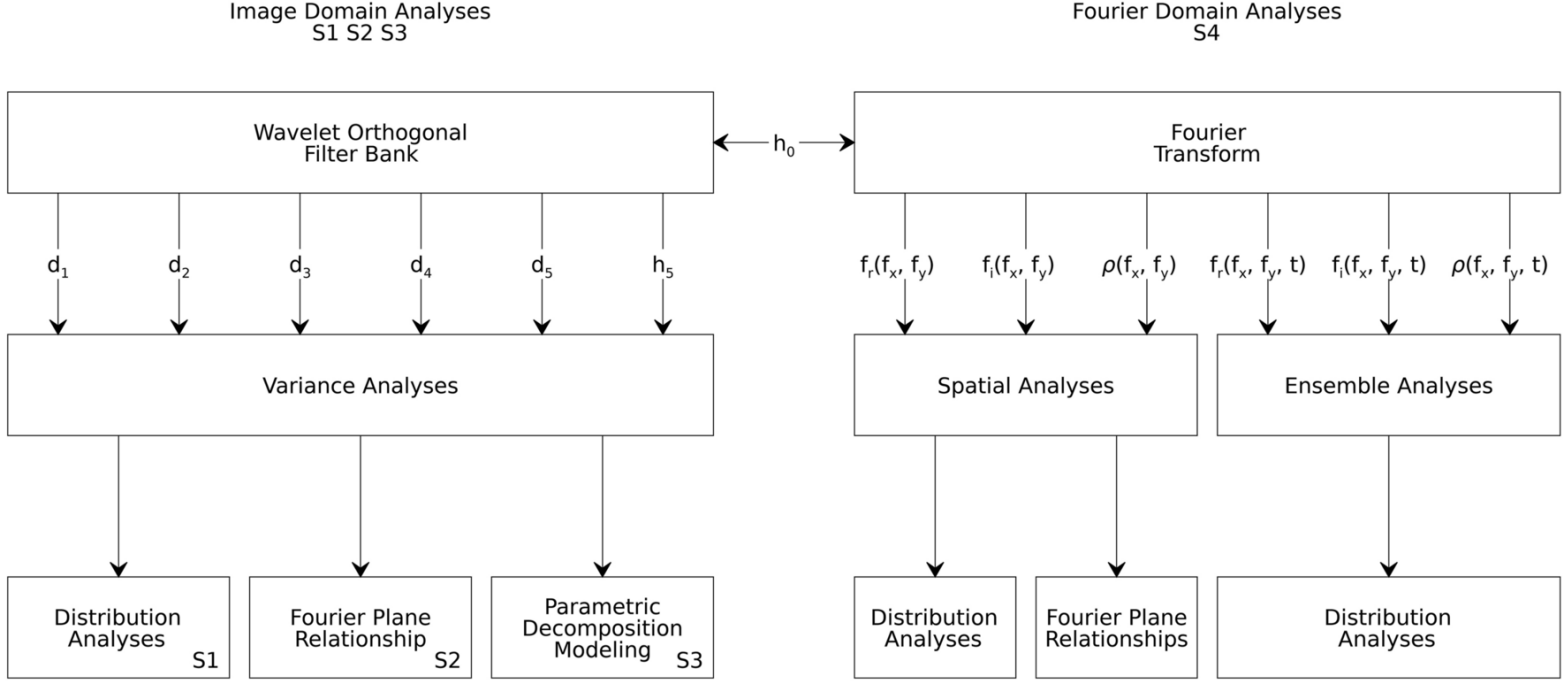
Investigation flow chart: this shows the overall planned investigation. The four strategies (S1, S2, S3, and S4) have been identified as well. On the left-side, h_0_ is analyzed with the wavelet transform (forward and disaggregated inverse); this is equivalent to an input into an *orthogonal filter-bank* that outputs 6 orthogonal filtered images (d_1_–d_5_, and h_5_) whose sum produces h_0_. On the right side, the Fourier transform was taken of each image producing its real and imaginary components and power spectrum [i.e., f_r_(f_x_, f_y_), f_i_(f_x_, f_y_), and ρ(f_x_, f_y_), respectively, referred to as the Fourier constituents]. When considering all images from a given experiment as an ensemble, the index t was introduced to the respective Fourier coordinates to account for the realization ordering.

Part of this study was constrained to making comparisons in the ID using the wavelet expansion of WN and comparing it to SDN. Another part of this study was constrained to characterizing the FD properties of WN for comparisons. Figure 1 presents the parallel investigation plan with essentially two separate pathways (ID or FD) for a given input image defined as h_0_. SDN was analyzed in the same manner as WN for comparisons. Although the analysis plan shows distinct paths, the characteristics are not disjoint but coupled by the FD.

In the ID study (left-side of Fig. 1), h_0_ undergoes a wavelet transform (forward and then disaggregated inverse). This is equivalent to an input into an *orthogonal filter bank* that outputs 6 filtered images (i.e., expansion images) whose sum also produces h_0_. The variances from all images were analyzed in several capacities: *distribution analysis*, where the variance distributions from the expansion images were summarized; *parametric modeling of the variance decomposition*, where the variances were theoretically modeled as a function of their expansion index; and *spectral distribution illustrations,* where an idealized Fourier domain plane layout was used to show the spectral spatial frequency cutoffs for the expansion images. These idealized cutoffs were then compared with empirical measurements. This part of the plan couples the two pathways shown in Fig. 1.

In the FD study (right-side of Fig. 1), h_0_ undergoes a Fourier transform (FT) that produces its complex (i.e., real and imaginary) components that are then used to calculate the power spectrum for each image; these three entities define the FD constituents in this paper. These were treated as *images* for a lack of better terminology. Spatial analyses that were applied to these images included the following: *distributional analyses,* where the mean and variance distributions were summarized across the Fourier plane; and *parametric modeling*, where adding the real and imaginary parts of a given FT quadratically was treated as a random variable transform, and the resulting pdf was analyzed parametrically (comparing predicted with empirical pdfs); and *ensemble analyses*, where the mean and variance distributions for each type of noise were investigated.

Specific definitions and conventions used in this paper that may vary across authors are provided. The power spectrum (or equivalently spectral density) of a given image is defined as the magnitude squared of its FT. The FT routine used here conserves power across domains without scaling constants. The variance of a mean centered image is equivalent to summing over the discrete points of the power spectrum in the FD. The lag-autocorrelation function and power spectrum are FT pairs for a given image within a scaling constant. For ease of notation, we used loose (*or not proper*) definitions for a delta and unit impulse functions. Signals analyzed here were both finite and discrete in both domains, whereas some derivations assume continuous variables. A signal that has an extremely large value at one location with a finite area is labeled as a delta function in specific contexts. For example, if a random signal has a uniform power spectrum over a finite frequency range and is zero elsewhere, the inverse FT produces the signal’s lag-autocorrelation function. In the discrete FT case, this inverse Fourier relation would produce a sinc-function (possibly scaled) evaluated at its zero-crossings. This gives zero correlation everywhere except at zero-lag. For our purposes in general, this signal behaves like a scaled delta function (or scaled unit impulse). For reference, the existence of Fourier pairs that are approximately truncated in both domains are discussed by Slepian^64^. WN is defined as a zero-mean normally distributed random noise image without spatial correlation. The expectation operator was used for both spatial (from a given image) and ensemble measures; the usage was made clear when applied. When referring to SDN, we implicitly mean the type used in this investigation, unless specified otherwise. The 1000 WN images assume the role of the 2D analog of the 1D time invariant stochastic system, described above. Various predictable properties of this process were developed as standards for comparisons.

### Study data and normalizations

Screening mammograms (n = 1000 images from different women) without breast cancer were used for this study. Images were acquired with either Hologic (Hologic Inc., Marlborough, Massachusetts) Selenia full field digital mammography 2D units or Hologic Dimensions digital breast tomosynthesis units (operating in the 2D mode). Mammograms have 70 µm pitch with 12-bit dynamic range per pixel and were acquired with two sizes used in normal operating conditions: 3328 × 4096 pixels for large compression paddle images and 2560 × 3328 pixels for small compression paddle images. For reference, in clinical images adipose tissue appears darker and fibroglandular lighter.

Two types of images were used to construct SDN, both functionally defined as s(x, y), using these factors: *synthetic* mammograms defined as g(x, y) or mammograms defined as m(x, y). SDN images were constructed with the multiplicative model expressed as

5a $$\text{s}\left(\text{x},\text{y}\right)=\text{g}\left(\text{x},\text{y}\right)\times \text{n}\left(\text{x},\text{y}\right)$$

or

5b $$\text{s}\left(\text{x},\text{y}\right)=\text{m}\left(\text{x},\text{y}\right)\times \text{n}\left(\text{x},\text{y}\right).$$

Power spectra for g(x, y) were constructed to obey a $\frac{1}{{\mathbf{w}}^{3}}$ power law, where **w** is a radial spatial frequency variable with $\upbeta$ = 1.5. Briefly, 2D random noise images were filtered in the FD (by multiplication) followed by Fourier inversion to produce g(x, y)^65^. This process was used to generate 1000 images. Different realizations of g(x, y) and n(x, y) were used for each s(x, y) construction. Additionally, 1000 SDN images were generated with m(x, y). Different m(x, y) and n(x, y) realizations were also used for each s(x, y) construction. Both n(x, y) and g(x, y) were generated with double precision. When considering n(x, y) or s(x, y) formed with g(x, y), n(x, y) and g(x, y) were generated with 2000 × 2000 pixels. When constructing s(x, y) with m(x, y), a given n(x, y) was generated with the same pixel dimensions as its respective m(x, y) factor. Manipulations and findings from n(x, y) alone are referred to Experiment 1. In a similar manner, experiments 2 and 3 refer to manipulations that used g(x, y) and m(x, y) respectively. For ensemble analyses, where necessary, t was introduced to index the ordering of the 1000 images in a given experiment [e.g., n(x, y, t)]. One random sample from each experiment was selected and used throughout the presentation for illustrations referred to as samples 1, 2 and 3, respectively (i.e., defined in accord with the definitions of the three experiments).

Experiments 2 and 3 can be considered as adding successive perturbations to Experiment 1. Experiment 1 is a stationary ensemble (either within or across images), where each image is a different realization from the same random process. Likewise, Experiment 2 introduced g(x, y) as a perturbation to Experiment 1 by disturbing its spatial uniformity. A given g(x, y) is a statistical realization of the same random process even though it may appear similar to mammograms. It follows, each g(x, y) × n(x, y) is also a realization from the same random process (i.e., a commonality with Experiment 1). Experiment 3 can be viewed as producing a random process by adding an incremental perturbation to Experiment 2 by switching *synthetic mammograms* with mammograms, but the resulting ensemble cannot be assumed stationary due to varying m(x, y). Studying how these images transform may shed light on the differences between WN and the two types of SDN.

**Fig. 2:**
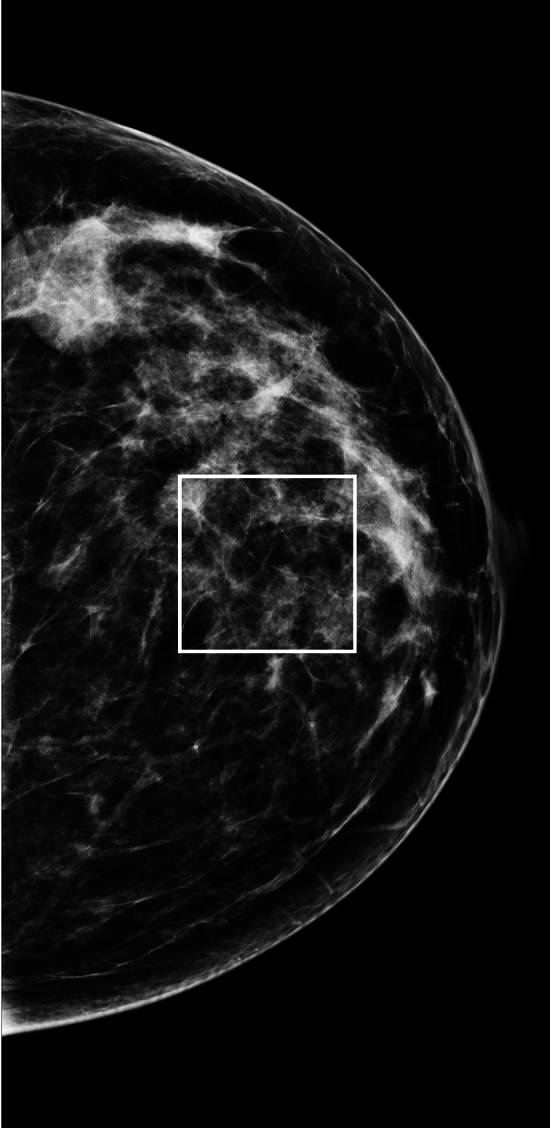
Mammogram region of interest illustration: this shows a mammogram selected at random with the centrally located 500 × 500 pixel region of interest determined with an automated algorithm.

All image dimensions were kept the same size in pixels, making the final analysis dependent upon possible size limitations of m(x, y). Moreover, Fourier analyses requires a rectangle that includes only the signal for precise calculations making dimension limitations dependent on m(x, y). An automated preprocessing check prior to the selection of m(x, y) in our dataset indicated we could fit a 500 × 500 pixel region of interest (ROI) approximately in the central breast area using these selection criteria: without including off breast area; without eliminating too many images; and keeping the ROI position within the area that approximates the *constant* compressed breast thickness region. These choices were a tradeoff, as we favored including more m(x, y) samples with less area than fewer samples with greater area for increased variation purposes. Figure 2 shows an ROI positioned automatically within the random selection from Experiment 3 (i.e., sample 3). This ROI is relatively small considering the breast size but is not representative of the entire dataset due to the random selection and breast size correlates with the compression paddle size. Wavelet expansions were applied to the entire mammogram and then this ROI was extracted. Hereafter, we refer to the respective ROIs as m(x, y). The other images were prepared similarly before cropping to 500 × 500 pixels and are also referred to as g(x, y) and s(x, y).

White noise images were generated with the Box-Muller method and investigated. Each WN image was decomposed into its orthogonal components based on the wavelet expansion (J = 5). This technique was used to investigate the variance decomposition as a function of the expansion images’ index and examine their related spectral spatial distributions and bandpass cutoffs in the FD. These images were generated first with 2000 × 2000 pixels. All n(x, y) were z-transformed first (mean centered and unit variance) and then multiplied by $\sqrt{\text{16,000}}$ giving a variance = 16,000 (an arbitrary normalization). The z-transform was applied such that the mean and variance were calculated from a 500 × 500 pixel ROI located near the geometrical center of the respective larger image. This ROI was then extracted after the wavelet expansion and used as the image, referred hereafter to as either n(x, y). SDN images were normalized and cropped in the same manner while also retaining their names. Results are presented as summaries from the respective distributions. These procedures eliminated wavelet filtering outside edge effects and ensured that the ROIs had the *same* variance and were identically zero mean as required to standardize the Fourier spectral analyses. Although the z-transform ensured the ROIs were identically zero-mean and unit variance, the convolutions in the wavelet transform can perturb these quantities in m(x, y).

### Wavelet expansion and image domain variance decomposition

The wavelet algorithm and theory used in this paper were described by Daubechies^66^. Our work builds on a bandpass orthogonal filter bank description of the ID wavelet expansion^65,67–69^. Although this approach was used in prior work, the objectives were different. Previously, the expansion was used for first order probability modeling in the ID to develop normal tissue detection methods, and to show similarities between disparate images by splitting them into their more regular components and more statistically irregular components (explained below). The bandpass approach was also used to develop an algorithm to characterize noise variation as a function of the signal non-parametrically for SDN research^28^. For the most part, this orthogonal sum description leaves the transform mechanics out of the developments, while illustrating several of the wavelet expansion’s inherent characteristics. The properties stated below are fundamental to wavelet analysis, but the related spectral distribution for WN and its variance decomposition in the ID may not be directly apparent.

To address Strategy 1, the ID variance decomposition based on the wavelet expansion was investigated. The expansion is as a complete sum of orthogonal images (i.e., their pairwise inner product expectation vanishes when considering different expansion images) that decompose a given image based on spatial scale in a specific manner discussed below. The expansion image approach is a disaggregated inverse wavelet transform, where the number of component images in the sum can be *user defined* to a limit. In the forward wavelet transform, the image size is successively reduced by a factor of ½ in each spatial dimension relative to its input size at each decomposition level limiting J. Wavelet coefficients (defining the wavelet domain) are produced by convolutions (separable 1D high and low pass filter operations applied in each dimension) in these reduced sized images. As the reduced sized image approaches the wavelet filter kernel length, it is prudent to terminate the expansion level J. Expansion images can be considered as the outputs of an orthogonal bandpass filter bank, where their sum reproduces the input image *exactly.* A first level, J = 1, expansion gives two orthogonal outputs

6 $${\text{h}}_{0}={\text{d}}_{1}+{\text{h}}_{1},$$

where h_0_ is the input image. Conversely, d_1_ and h_1_ are the high and low half bandpass output images, respectively. All images are implicit functions of spatial coordinates (x, y). In Eq. (6), E[h_0_] = E[h_1_] because E[d_j_] = 0 (by definition), where E[**z**] is the expectation operator, referenced earlier, applied to **z**. Here, this operator is used to derive either spatial summaries from a given image or quantities from distributions unless noted otherwise. The d_1_ image contains the detail lost as h_0_ is blurred to its next coarse version of itself: h_1_ = h_0_ − d_1_. Taking the expectation of the inner product of both sides of Eq. (6) and applying the expansion image orthogonality conditions gives

7 $${\text{E}[\text{h}}_{0}^{2}] =\text{ E}[{\text{d}}_{1}^{2}]+{\text{E}[\text{h}}_{1}^{2}] = {\upsigma }_{1}^{2}+{\text{E}[\text{h}}_{1}^{2}],$$

where ${\upsigma }_{1}^{2}$ is the variance of d_1,_ and the cross terms vanished. Squaring the mean of h_0_ and subtracting it from both sides of Eq. (7) gives the first level expected variance decomposition

8 $${\text{v}}_{0}^{2}= {\upsigma }_{1}^{2}+ {\text{v}}_{1}^{2},$$

where ${\text{v}}_{0}^{2}$ is the variance of h_0_, and ${\text{v}}_{1}^{2}$ is the variance of h_1_. Decomposing h_1_, gives an *identical* expression

9 $${\text{h}}_{1}={\text{d}}_{2}+{\text{h}}_{2},$$

where d_2_ is the high half band of h_1_, and h_2_ is the low half band. Because E[d_2_] = 0, it follows E[h_1_] = E[h_2_]. Equivalently, the d_2_ image is the output of a bandpass filter that blocks the lower quarter frequency band and the high half band of h_0_ or blocks the lower half band of h_1_. Similarly, the filter outputs can be summed to reproduce h_1_. The same orthogonality and variance conditions apply to Eq. (9). Because E[d_1_ × d_2_] = 0, it follows E[d_1_ × h_2_] = 0 from Eqs. (6) and (7) [or, by definition]. Substituting Eq. (9) in Eq. (6), applying Eq. (7), and subtracting the mean-squared from both sides gives an equivalent expression for Eq. (8): ${\text{v}}_{0}^{2}= {\upsigma }_{1}^{2}+{\upsigma }_{2}^{2}+ {\text{v}}_{2}^{2}$, where the additional new terms are the variances of d_2_ and h_2_ respectively. More generally, h_j-1_ = d_j_ + h_j_, where d_j_ is the detail lost as h_j-1_ is blurred to h_j_, and j is an integer index ranging from 1 to J. Because E[d_j_] = 0, it follows E[h_0_] = E[h_j_] = E[h_J_]. Continuing the iteration gives the filter bank outputs that can be summed to reproduce the input image

10 $${\text{h}}_{0}={\text{h}}_{\text{J}}+{\sum }_{\text{j}=1}^{\text{J}}{\text{d}}_{\text{j}}.$$

The d_j_ images (i.e., detail images or the more regular components) are bandpass filtered versions of the input image, and h_J_ is a coarse version of h_0_ (i.e., the irregular component for mammographic or natural scene type images), where the degree of coarseness depends on the terminal value of J. Equation (10) illustrates the wavelet scale property. As j increases, the image structure captured in d_j_ tends to longer range features (i.e., lower spatial frequencies) as does h_J_. Continuing the process, by substituting Eq. (10) in Eq. (6), taking the inner product expectations, using the orthogonality conditions, and removing influence of the mean, produces the ID variance decomposition expression that parallels Eq. (10)

11 $${\text{v}}_{0}^{2}= {\text{v}}_{\text{J}}^{2}+ \sum_{\text{j}=1}^{\text{J}}{\upsigma }_{\text{j}}^{2},$$

where ${\upsigma }_{\text{j}}^{2}$ is the variance of d_j_ and ${\text{v}}_{\text{J}}^{2}$ is the variance of h_J_.

A given d_j_ image can be decomposed further into three orthogonal component images as well giving

12 $${\text{d}}_{\text{j}}{=\text{d}}_{\text{jh}}+{\text{d}}_{\text{jv}}+ {\text{d}}_{\text{jd}},$$

where the h, v and d indices define the horizontal, vertical, and diagonal components at a given scale. In this convention, the subscript h indicates the wavelet operations were performed to capture striations and linear structures along the x-direction (high pass filter applied in the y-direction), the subscript v to capture these features along the y-direction (high pass filter applied in x-direction), and subscript d to capture features with signatures in both directions. In the FD at a given scale, d_jh_ will have a stronger signature in the high spatial frequency range in the y-direction and weaker signature in the lower spatial frequency range in the x-direction. Likewise, d_jv_ will have a stronger signature in the high spatial frequency range in the x-direction and weaker signature in the lower spatial frequency range in the y-direction. The diagonal component (high pass in both directions) will have a strong signature in the high spatial frequency range in both the x- and y-directions in the FD and is labeled as d_jd_. The variance of d_j_ is the sum of the variances of its components (not developed theoretically further in this paper); these components give a more complete description of the FD view. The decomposed variance distributions were compared within and across experiments.

The wavelet transform algorithm (forward and reverse transforms) used in this investigation, and related work^27,28,46,47,49,51,52,65,67–70^, was developed by one of the authors (JH) in the C programming language. This algorithm works with conventional wavelet bases and operates on gray-scaled 2D images of arbitrary dimensions without zero-padding the input. The wavelet basis used here has 12 coefficients^26^. This basis is nearly symmetric, is often referred to as a symlet, and has become the standard for our wavelet research to date.

### Wavelet expansion and Fourier plane relationships

**Fig. 3:**
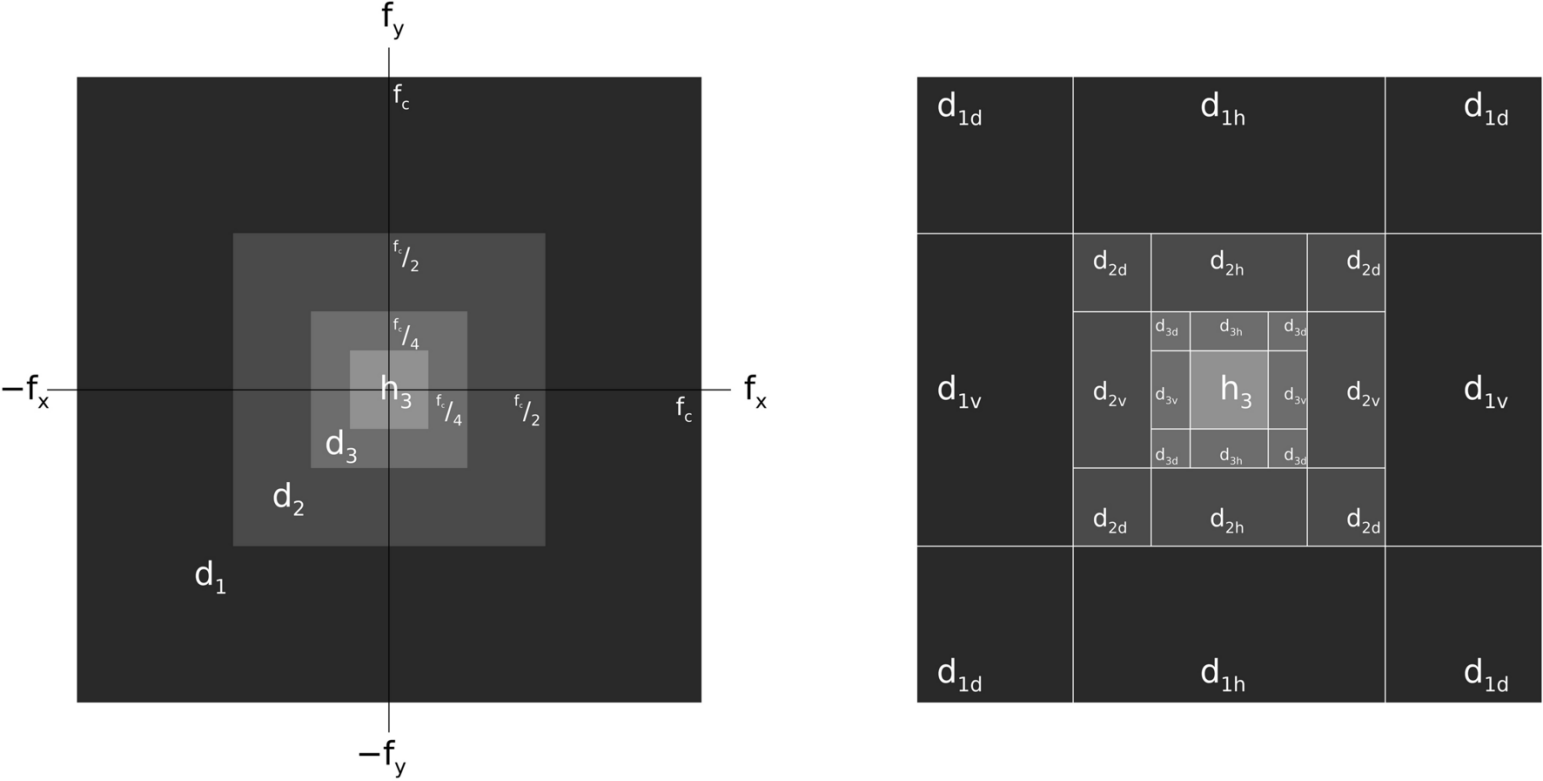
Fourier plane spectral density bandwidth regions for the expansion images: the left pane shows the spectral cutoffs for a J = 3 wavelet expansion. The right pane shows the same regions further subdivided, where each d_j_ is expanded into its horizontal, vertical, and diagonal components. The DC component is in the center of the plane.

To address Strategy 2, the Fourier transform (FT) of the expansion images is illustrated. The Fourier plane is shown in Fig. 3 (left) for a J = 3 expansion with an idealized overlay (limited to J = 3 due to graphical labeling size constraints). The related power spectra bandpass cutoffs are described (i.e., boundaries) for the expansion images. Fourier domain Cartesian coordinates are expressed as (f_x_, f_y_), where f_x_ and f_y_ are the respective spatial frequency coordinates in the x- and y-directions with (0, 0) in the center of the plane. The real and imaginary parts of the FT are defined as f_r_(f_x_, f_y_) and f_i_(f_x_, f_y_) respectively (or equivalently as f_r_ and f_i_ for short, where $\sqrt{-1}$ factor is incorporated into the f_i_ term). This coordinate system is used in all the Fourier plane illustrations in this article, although not labeled hereafter. We define |f_c_| as the highest resolvable Cartesian spatial frequency component in the image, considered isotropic in both spatial frequency directions. If we consider a perfect cutoff filter, the d_1_ spectrum is constrained to the outer dark region. The inner boundary intersects the spatial frequency coordinate axes at these points: ($\frac{{\text{f}}_{\text{c}}}{2}$, 0), (0, $\frac{{\text{f}}_{\text{c}}}{2}$), ($\frac{-{\text{f}}_{\text{c}}}{2}$, 0) and (0, $\frac{-{\text{f}}_{\text{c}}}{2}$). The outer boundary intersects the coordinate axes at these points: (f_c_, 0), (0, f_c_), (-f_c_, 0), and (0, -f_c_). The region corresponding to d_1_ covers ¾ of the frequency plane area, a key proportion used in the derivations below. Boundaries for the other d_j_ images follow the same pattern by replacing f_c_ with $\frac{{\text{f}}_{\text{c}}}{{2}^{\text{j}-1}}$ for the outer intersections and $\frac{{\text{f}}_{\text{c}}}{{2}^{\text{j}}}$ for the inner intersections. In Fig. 3 (left), the spectrum of h_3_ is constrained to the interior square about the origin. As J increases, the same pattern continues sandwiching the spectrum of h_J_ to a smaller and smaller region about the origin. The d_j_ images with smaller j account for wider frequency bands. Figure 3 (right) shows the Fourier plane view with the d_j_ directional decomposition. Idealized perfect cutoff filters were used in this FD illustration that show the wavelet expansion is equivalent to performing an octave sectioning of the frequency plane (i.e., the specific manner referred to above). In practice, cutoff profiles vary with the number of wavelet coefficients. A wavelet with an increased number of coefficients will generally have sharper cutoff profiles in the FD. Variances in the d_j_ and h_J_ images were compared to these conceptual diagrams. This depiction of the wavelet expansion is its equivalent description in the FD.

### Parametric modeling of the variance decomposition

For Strategy 3, the variance decomposition of n(x, y) was parametrically modeled as a function of its expansion image index. This was achieved by integrating over the wavelet bandpass regions in the FD and applying Fourier power conservation laws. For a given image, the power spectrum is defined as: ρ(f_x_, f_y_) = ${\text{f}}_{\text{r}}^{2}+ {\text{|f}}_{\text{i}}|^{2}$. A normalized Fourier coordinate system was used paralleling Fig. 3, where |f_c_|= 1. Consequently, the Fourier plane covers 4 × [spatial frequency]^2^ units. Starting with a J = 1 level expansion, the total expected power for h_1_ is given by

13 $${\text{v}}_{1}^{2}=\text{ E}\left[ {\int }_{0}^\frac{1}{2}{\int }_{0}^\frac{1}{2}\uprho \left({\text{f}}_{\text{x}}, {\text{f}}_{\text{y}}\right){\text{df}}_{\text{x}}{\text{df}}_{\text{y}} \right]= {\text{v}}_{0}^{2}\times \frac{1}{4},$$

where $\uprho ({\text{f}}_{\text{x}}, {\text{f}}_{\text{y}})$ is the statistically uniform power spectrum of n(x, y) with a quarter of its variance accounted for in the lower half of the upper right quadrant. The integrand in Eq. (13) was multiplied by 4 to account for the power in all four quadrants and divided by 4 to normalize for the Fourier plane area; the integration was valid in the Riemann–Stieltjes sense^63^. To conserve power, the expected variance for d_1_ follows: ${\upsigma }_{1}^{2} ={\text{v}}_{0}^{2}\times \frac{3}{4}$. The rest of the chain was deduced from these proportions as three quarters of the remaining variance is accounted for in d_j_. The expected variance for d_2_ is given by

14 $${\upsigma }_{2}^{2}={\text{v}}_{0}^{2}\times \frac{3}{4 }\times \frac{1}{4}={\text{v}}_{0}^{2}\times \frac{3}{16}.$$

It follows, the parametric expression for the expected variance of d_j_ is given by

15 $${\upsigma }_{\text{j}}^{2}=\frac{3{\times \text{v}}_{0}^{2}}{4}\times (\frac{1}{4}{)}^{\text{j}-1}.$$

Equation (15) shows the variance attenuates exponentially as a function of increasing j (i.e., j = 1 to J), or equivalently, as a decreasing function of spatial frequency bandpass location. The index j relates the expansion image variance to the related FD regions shown in Fig. 3 in the left-panel. Taking log_2_ of both sides of Eq. (15) gives

16 $${\text{log}}_{2}\left({\upsigma }_{\text{j}}\right)={\text{log}}_{2}\left({\text{v}}_{0}\right)+{\frac{1}{2}\times \text{log}}_{2}\left(3\right)-\text{j}=-\text{j}+\text{B},$$

where B is the collection of constants. Equation (16) is a linear function of j relating the standard deviation of d_j_ with the standard deviation of the input image. The validity of Eqs. (15) and (16) were first investigated with WN using Eq. (16). When established, Eq. (16) was used as the reference to model the variance decomposition of SDN in the same manner. The indices i = 1, 2, and 3 were used to distinguish the findings from each experiment: M_i_ and B_i_, for example.

### Fourier domain analyses

Background material on SDN is required to make clear the importance of Strategy 4 (right-side of Fig. 1). In the FD, SDN images can be considered as outputs from a filtering process. We use **F**[**q**] to define the 2D FT operation on the arbitrary image **q**. Using the Fourier multiplication-convolution pair property, **F**[s(x, y)] is equivalent to the 2D convolution of **F**[n(x, y)] with either **F**[m(x, y)] or **F**[g(x, y)]. It follows, **F**[g(x, y)] and **F**[m(x, y)] can be considered as filter kernels in the FD. The arguments used above to describe the ID perturbations follow in the FD as well. When constructed with g(x, y), **F**[s(x, y, t)] are statistical realizations of the same complex random process for each t, although the spectra are similar to mammograms. When constructed with m(x, y), it cannot be assumed that **F**[s(x, y, t)] are complex statistical realizations of the same random process for each t (i.e., also acting as a perturbation to the Experiment 2 ensemble). It is worth noting, research shows mammograms are nonstationary in the ID^71^.

For Strategy 4, the FD properties of WN (i.e., h_0_) were described as comparators for SDN. The real and imaginary components, as well as spectra, were treated as *images* to investigate uniformity across the Fourier plane by both physical observations and analyses of the means and variances. The complex amplitudes at (f_x_, f_y_) were also treated as random variables, added quadratically to form the power spectrum, and determine its pdf; normalized histograms (empirical pdfs) were used as pdf approximations (referenced as pdfs).

Known properties of **F**[n(x, y, t)] were developed for comparison purposes. In this section, the expectation operations imply ensemble quantities or metrics derived from pdfs. These properties for WN follow the 2D spatial analyses. The FT is a *unitary* linear transformation. **F**[n(x, y, t)] produces zero mean joint normal independent identically distributed random variables at each complex point^72^. We then define $\frac{{\upsigma }_{\text{n}}^{2}}{2}$ as the expected ensemble variance at either f_r_(f_x_, f_y_) or f_i_(f_x_, f_y_). The average FD spatial variance from a given image can by expressed in terms of the expected ensemble power at ρ(f_x_, f_y_). This is achieved by applying the Fourier power conservation law across domains, using the definition of ρ(f_x_, f_y_) given above in Section "Parametric modeling of the variance decomposition", and that **F**[n(x, y, t)] produces joint normal variates as mentioned above^72^. For an FT with n^2^ points, it follows in this case: ${\upsigma }_{\text{n}}^{2}\times {\text{n}}^{2}=\text{16,000},$ E[ρ(f_x_, f_y_)] = ${\upsigma }_{\text{n}}^{2}=0.064$, and either ρ(f_x_, f_y_), or ρ(f_x_, f_y_, t), is distributed exponentially as ${\text{g}}_{1}\left(\uprho \right)= \frac{1}{\text{k}}\times \text{exp}\left(-\frac{\rho }{\text{k}} \right).$ Both the mean and standard deviation are given by ${\text{k}=\upsigma }_{\text{n}}^{2}$. Taking the natural logarithm of ${\text{g}}_{1}\left(\uprho \right)$ gives

17 $$\ln \left( {{\text{g}}_{1} \left( {\uprho } \right)} \right) = - \ln \left( {\text{k}} \right) - \frac{{\uprho }}{{\text{k}}}.$$

Equation (17) is linear with the slope inversely related to k. The empirical pdf for k was investigated with regression analysis to find the slope ≈ -1/k [applied to each ρ(f_x_, f_y_ )]. The intercept, in general, will not be equal to -ln(k) without conditioning g_1_ because the dynamic range of ρ could be restricted and histogram binning. In the analysis of Eq. (17), ensemble statistics were switched with their respective spatial statistics. This switch is valid for the collection of n(x, y) because Experiment 1 is stationary in both domains. Each **F**[n(x, y)] was analyzed separately and results summarized. The fidelity of the empirical relationships with Eq. (17) was assessed. The empirical pdf for k, defined as h_1_(k), was found by fitting Eq. (17) to the natural logarithm of g_1_(ρ) for each of the 1000 images. Fitted lines were also assessed with linear correlation coefficients and summarized. The same process was applied to the SDN pdfs [i.e., g_2_(ρ) and g_3_(ρ)] to find the respective k-pdfs defined as h_2_(k) and h_3_(k). The k-distributions were compared with the Kolmogorov–Smirnov (KS) test and their distribution means with the t-test both applied at the 5% significance level. As above, k was given an index (k_i_) for i = 1, 2, or 3 to distinguish the findings from each experiment, respectively.

Ensemble FD analysis methods are described in this section (Fig. 1, on the left-side under Ensemble block). Because **F**[n(x, y, t)] is a stationary normally distributed complex ensemble^72^, a given FD ensemble statistic can be derived (spatially in this 2D study) from a single observation as above. Here, we examine ensemble expectations for comparisons with the spatial summaries. The expected means of ρ(f_x_, f_y_, t), f_r_(f_x_, f_y_, t), and f_i_(f_x_, f_y_, t) and variances of f_r_(f_x_, f_y_, t) and f_i_(f_x_, f_y_, t) were investigated. Here, expectations were taken over t for each FD coordinate. These operations produced 500 × 500 pixel images of a given ensemble quantity’s spatial distribution in the Fourier plane. These expected mean ensemble images are defined as: f_r_(avg); f_i_(avg); and ρ(avg) for the real, imaginary, and spectral constituents. The expected ensemble variance images are defined as: f_r_(var); and f_i_(var) for the real and imaginary components. Ensemble measurements in Experiment 1 were compared with the like measurements taken from experiments 2 and 3 [i.e., taken from s(x, y, t)]. The KS test was used to compare the respective ensemble pdfs from each image, and t-test to compare the ensemble distribution means, both at the 5% significance level. These were then compared with the spatial analyses performed in the FD (compared with the findings from fitting Eq. (17), specifically).

## Results

### Wavelet expansion and image domain variance decomposition

**Fig. 4:**
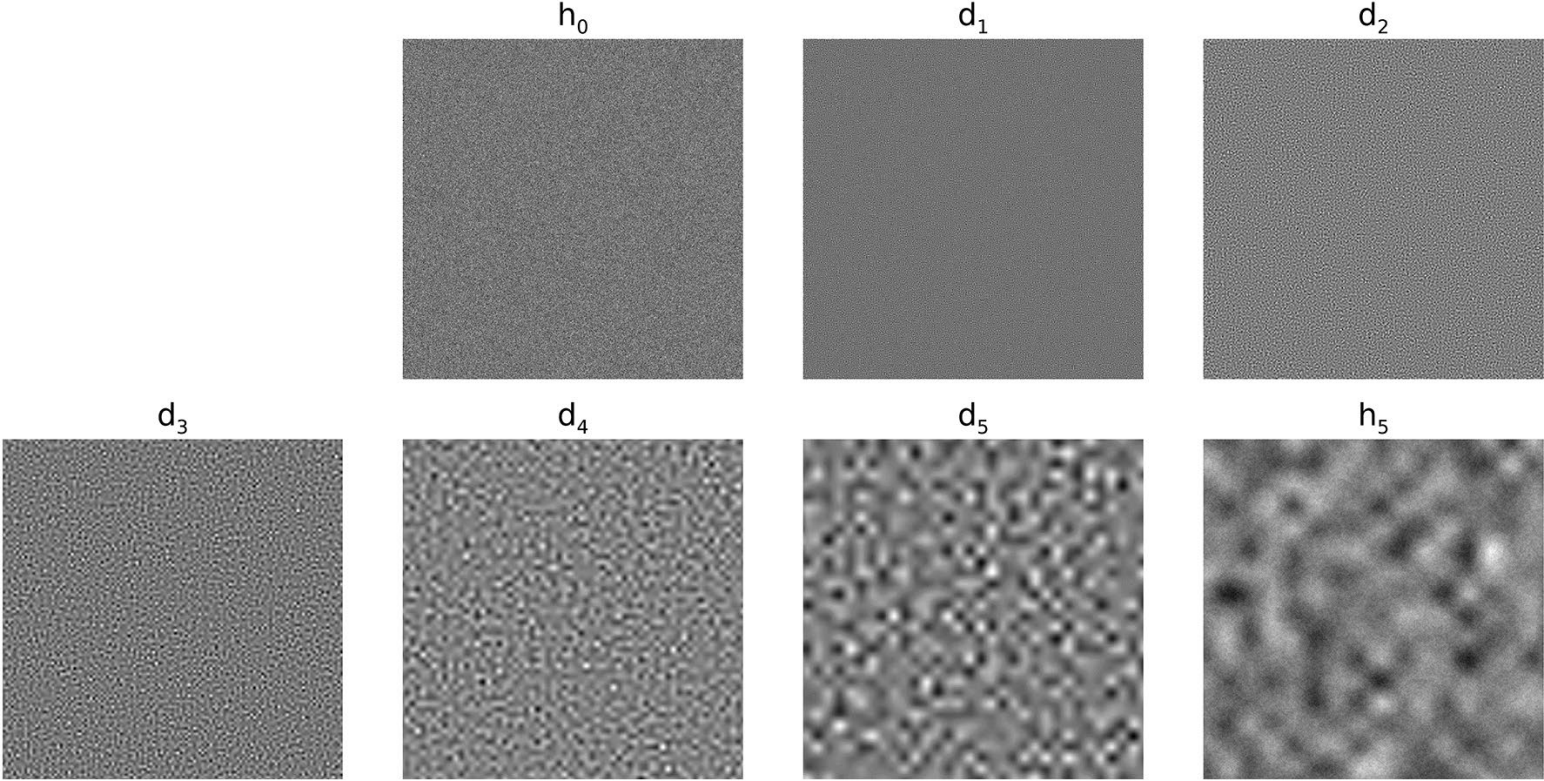
Wavelet expansion of sample 1: the 6 expansion images (d_1_–d_5_ and h_5_) for h_0_ (white noise)  are shown for a J = 5 decomposition. The texture changes from grainy to lumpy as j increases, and their sum reproduces h_0_.

**Fig. 5:**
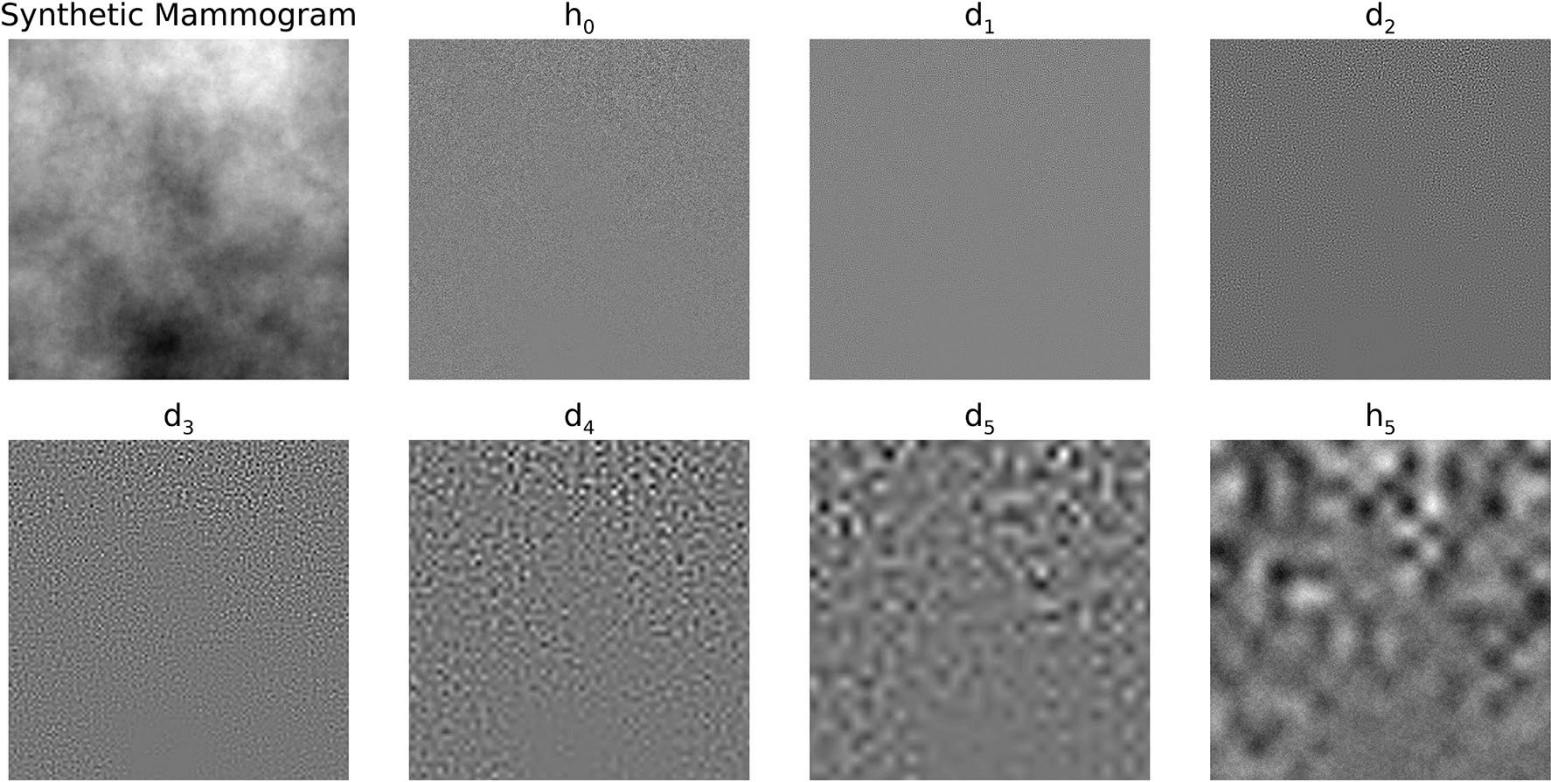
Wavelet expansion of sample 2: the 6 expansion images (d_1_–d_5_ and h_5_) are shown for h_0_ that is a (synthetic mammogram) × noise for a J = 5 expansion. Similarly, the texture changes from grainy to lumpy as j increases, and their sum reproduces h_0_.

**Fig. 6:**
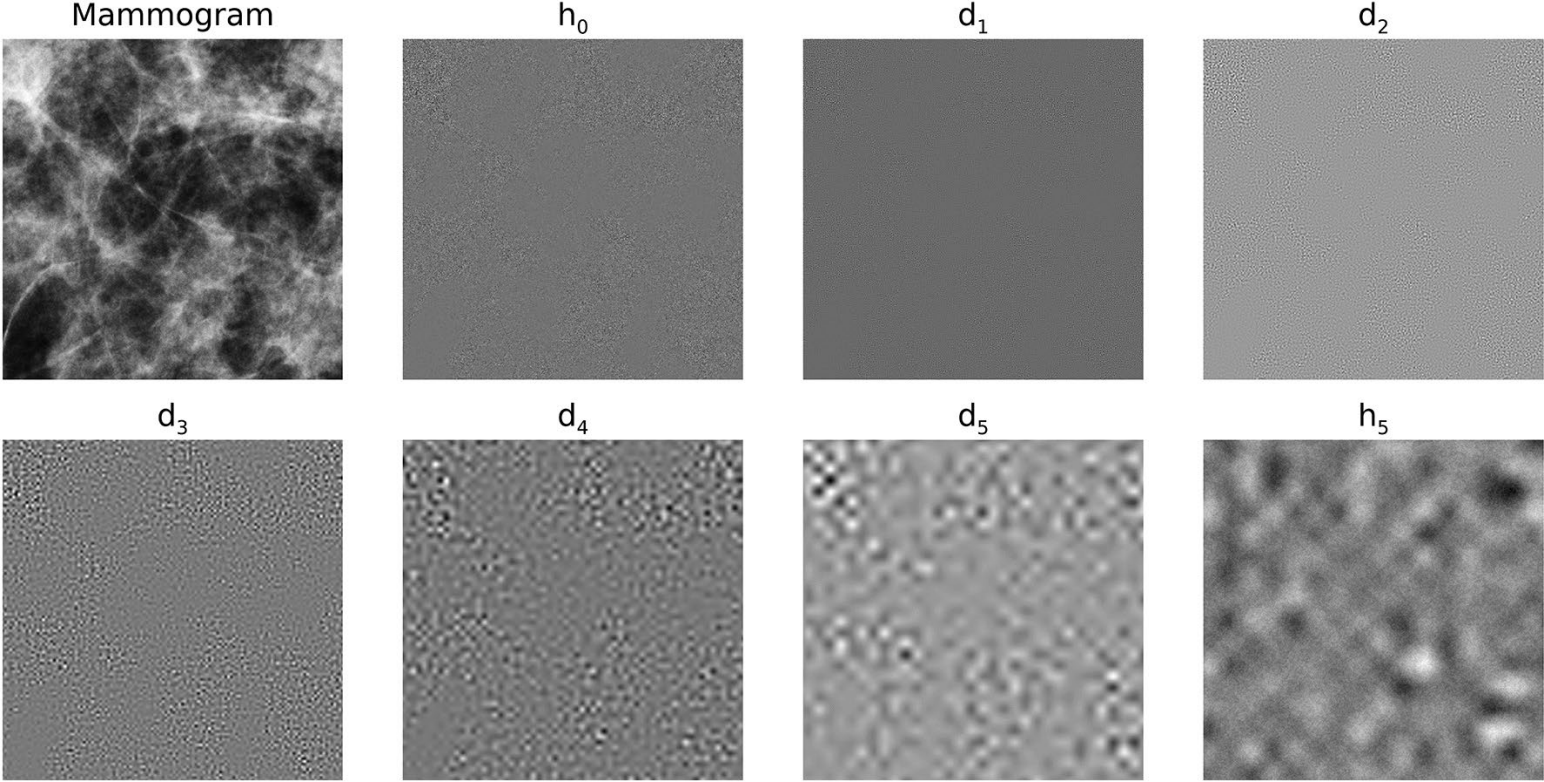
Wavelet expansion of sample 3: the 6 expansion images (d_1_–d_5_ and h_5_) are shown for h_0_ that is a mammogram × noise for a J = 5 expansion, where h_0_ is the region outlined in Fig. 2. Similarly, the texture changes from grainy to lumpy as j increases, and their sum reproduces h_0_.

To assess the merits of Strategy 1, the distribution summary findings from the wavelet expansion variance decomposition are shown in Table 1. This table gives the means and standard deviations for the h_0_ and d_j_ variance distributions. The variance means and standard deviations for each experiment are in close agreement when making comparisons across each row (i.e., across experiments for like d_j_ images); a given mean is relatively large relative to the respective distribution’s width. Variations across quantities in Table 1 are minor and likely induced by rounding in the wavelet expansion’s convolution operations and output pixel rounding. Visual illustrations of the expansion images for the sample from each experiment are shown in Figs. 4, 5 and 6, respectively. WN (Fig. 4) appears uniform without observable spatial variation for most of the expansion images in contrast with SDN images shown in Figs. 5 and 6. Nevertheless, the spatial scale property of the wavelet bandpass character is illustrated in each expansion level. As the bandpass shifts towards lower frequencies, the images contain longer spatial range characteristics (i.e., a lumpier characteristic) and vice versa (more grainy appearance).

**Table 1:** Summarized variance distributions for the wavelet expansion images.

Image	Experiment 1, mean (SD)	Experiment 2, mean (SD)	Experiment 3, mean (SD)
h_0_	16,000 (0)	16,000 (0)	16,000 (0)
d_1_	11,837 (20)	11,863 (31)	11,850 (25)
d_2_	2940 (17)	2946 (26)	2943 (22)
d_3_	725 (10)	728 (14)	725 (11)
d_4_	175 (5)	176 (7)	176 (6)
d_5_	42 (2)	42 (3)	42 (3)

These show the variance distributions’(n = 1000 for each experiment) means and the standard deviations (SDs), cited parenthetically, for each expansion image. The expected values of the variances are similar because of the wavelet exact reconstruction property and the controlled variance normalization boundary conditions superimposed on the images.

### Wavelet expansion and Fourier plane spectral relationships

**Fig. 7:**
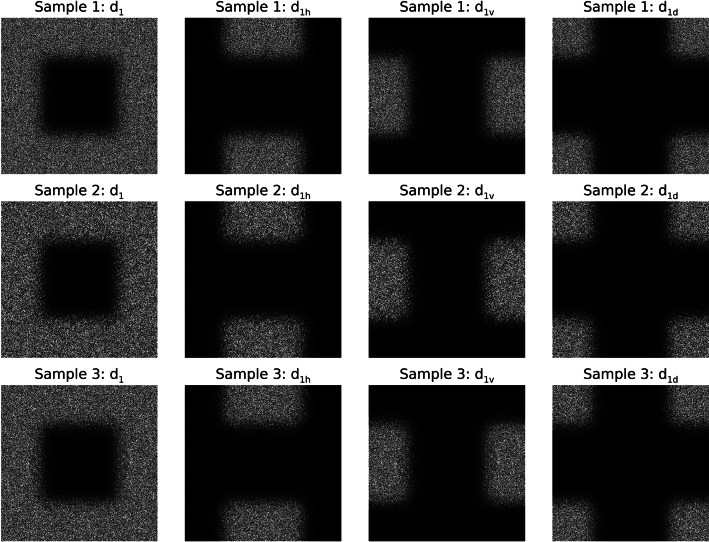
Fourier plane spatial distributions for the d_1_ power spectra for each sample: these show the Fourier plane for a J = 1 expansion for the samples (same Fourier coordinate system and layout Fig. 3). Each row shows the Fourier plane for a given sample. Each pane was contrasted for viewing purposes keeping the level and range the same across all panes. The total region corresponding to d_1_ is shown in the first column for each sample. The d_1_ images were then expanded into three components: d_1h_ in the 2nd column; d_1v_ in the 3rd column; and d_1d_ in the 4th column for each sample.

To assess the merits of Strategy 2, graphical illustrations of the wavelet expansion, viewed in the FD, are provided first as complementary evidence for uniformity. Fourier plane spatial distributions for a first level expansion are shown in Fig. 7 for the three samples. Window level and range were adjusted the same for these three illustrations. There are two important characteristics worth noting. First, these compare well with the conceptual FD layouts shown in Fig. 3 for a J = 1 expansion. Secondly, the chatter appears uniformly distributed *spatially* and is visually *similar* across each experiment. Moreover, there appears little difference in uniformity across the regions that account for the three components of d_j_ either within or across experiments. Illustrating expansions for greater J produced similar outputs and configurations in a plane reduced accordingly (i.e., change f_c_ to ½ × f_c_ for J = 2 and so on) and are not shown.

### Parametric modeling of the variance decomposition

**Fig. 8:**
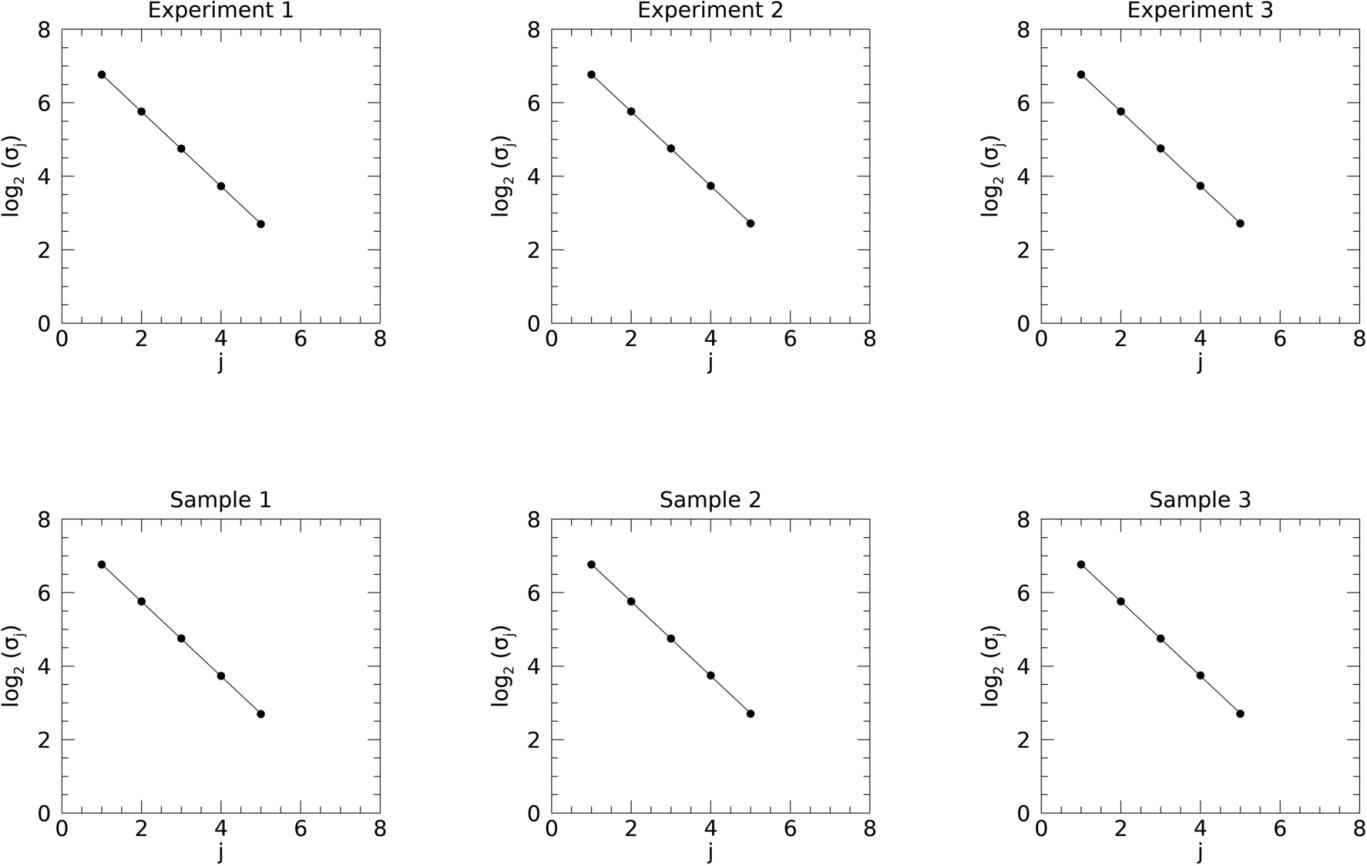
Parametric variance decomposition modeling for the wavelet expansion images: the top row used the respective variance distribution means from each experiment from Table 1. The logarithm of the expansion image standard deviation (σ_j_) is plotted (points) as a function of the expansion image index j (i.e., d_j_). Each experiment was modeled with the linear relationship (solid line) expressed in Eq. (16) with M_i_ for the slope and B_i_ for the intercept. Model parameters for the top row are provided with standard errors parenthetically: Experiment 1, E[M_1_] ≈ − 1.02 (0.0) and E[B_1_] ≈ 7.79 (0.01); Experiment 2, E[M_2_] ≈ − 1.01 (0.0) and E[B_2_] ≈ 7.79 (0.01); and Experiment 3, E[M_3_] ≈ − 1.02 (0.00) and E[B_3_] ≈ 7.79 (0.01). The bottom row shows the analogous plots in the same format for the point estimates derived from the samples: Sample 1, M_1_ ≈ − 1.02 (0.00) and B_1_ ≈ 7.79 (0.01); Sample 2, M_2_ ≈ − 1.01 (0.0) and B_2_ ≈ 7.79 (0.01); and Sample 3, M_3_ ≈ − 1.00 (0.0) and B_3_ ≈ 7.76 (0.03). In all plots, R ≈ − 1.0.

The merits of Strategy 3 were assessed by analyzing the regression plots shown in Fig. 8. The fitted parameters are compared with theoretical quantities predicted by Eq. (16). The top row gives the findings using the mean value from the respective σ_j_ distributions from each experiment. Related regression parameters are shown on the left-side of Table 2. The bottom row in Fig. 8 shows the point estimate plots for the samples, and the related regression parameters are shown on the right-side of Table 2. There is close agreement across the summaries from each experiment, as well as with the respective point estimates, approximated as: M_i_ ≈ − 1.01 and B_i_ ≈ 7.79 with R ≈ − 1.0 (indicating linearity). The standard errors (SEs) were approximately 0.01 for both M_i_ and B_i_ in all cases. Regression parameter estimates are in close agreement with the predicted values from Eq.^16^: M_p_ = − 1.0 and B_p_ ≈ 7.77. In all experiments, the variance for a given d_j_ image attenuated exponentially as a function of j *identically*. In sum, these illustrations and analytical findings for Strategy 3 showed the SDN images share common characteristics in both domains with WN: (i) the summarized power for the wavelet expansion images decreases exponentially as a function of the expansion index; and (ii) the variance for each expansion image is distributed uniformly over specified regions in Fourier plane.

**Table 2:** Wavelet expansion variance decomposition parametric modeling.

	E [M_i_]	E [B_i_]	M_i_ (pt)	B_i_ (pt)
Experiment 1	− 1.02 (0.0)	7.79 (0.01)	− 1.02 (0.0)	7.79 (0.01)
Experiment 2	− 1.01 (0.0)	7.79 (0.01)	− 1.01 (0.0)	7.79 (0.01)
Experiment 3	− 1.0 (0.0)	7.76 (0.03)	− 1.02 (0.0)	7.79 (0.01)

The left-side of the table gives estimates of the regression parameters (slope = M_i_, and intercept = B_i_) using the means from the variance distributions using Eq. (16) as the model. The right-side of this table gives the point (pt) estimates from the respective samples using the same model.

The developments and findings above demonstrate why the wavelet filter approach was *instrumental* for this investigation. The orthogonality conditions permitted the variance decomposition’s relatively simple parametric form. Likewise, the x–y separable 1D convolutions in the wavelet transform produce an output that renders itself useful for illustrating bandwidth cutoffs with Cartesian coordinates (i.e., square, or rectangular in form) in the FD when the starting point is a uniform power spectrum [see Figs. 3, 7, and Eq. (13)]. These conditions simplified the work considerably.

### Fourier domain analyses

**Fig. 9:**
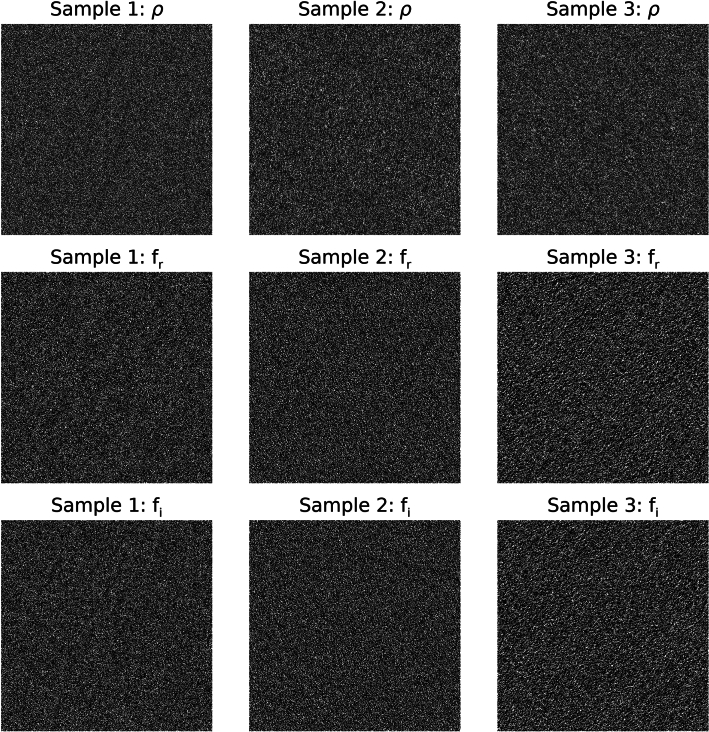
Fourier domain constituent sample images: top row shows the power spectra (ρ) for the samples selected from each experiment; the middle row shows the respective real parts (f_r_) of the Fourier transform (FT) for the samples; and the bottom row shows the respective imaginary parts (f_i_) of the FT for the samples. The same window level and range were used in all panes. The Fourier coordinate system is the same as that in Fig. 2. These are well approximated as statistically uniform (flat) chatter with intra row-wise comparisons.

To address the merits of Strategy 4, the FD images were analyzed. The sample images, shown in Fig. 9, are displayed with the same window level and range. Power spectra are shown in the top row and appear uniform with approximately the same mean ≈ 0.064. The respective distribution means were similar (~ 0.064) with parasitic SEs and SDs (~ 10^–5^ and 10^–4^ respectively). The real and imaginary parts of the FTs are shown in the middle and bottom rows, respectively with variances ≈ [0.032, 0.032] for each component for all samples. Expected variances for all experiments were about equivalent with variance distribution means: [0.032, 0.032]. The respective SEs and SDs were parasitic (~ 10^−7^ and 10^–5^, respectively).Although subjective, these findings suggest the real and imaginary components for the SDN appear to have somewhat different textures than WN (i.e., different spatial correlation) agreeing with derivations in the Appendix and operations performed on the three sample images (discussed and explored further below). A visual inspection of all FD images in each experiment by two of the authors indicated similar uniformity. Visual uniformity and narrow variance distribution widths for the real and imaginary components provide complementary evidence for spatial uniformity in the FD constituents for each of the three experiments.

**Fig. 10:**
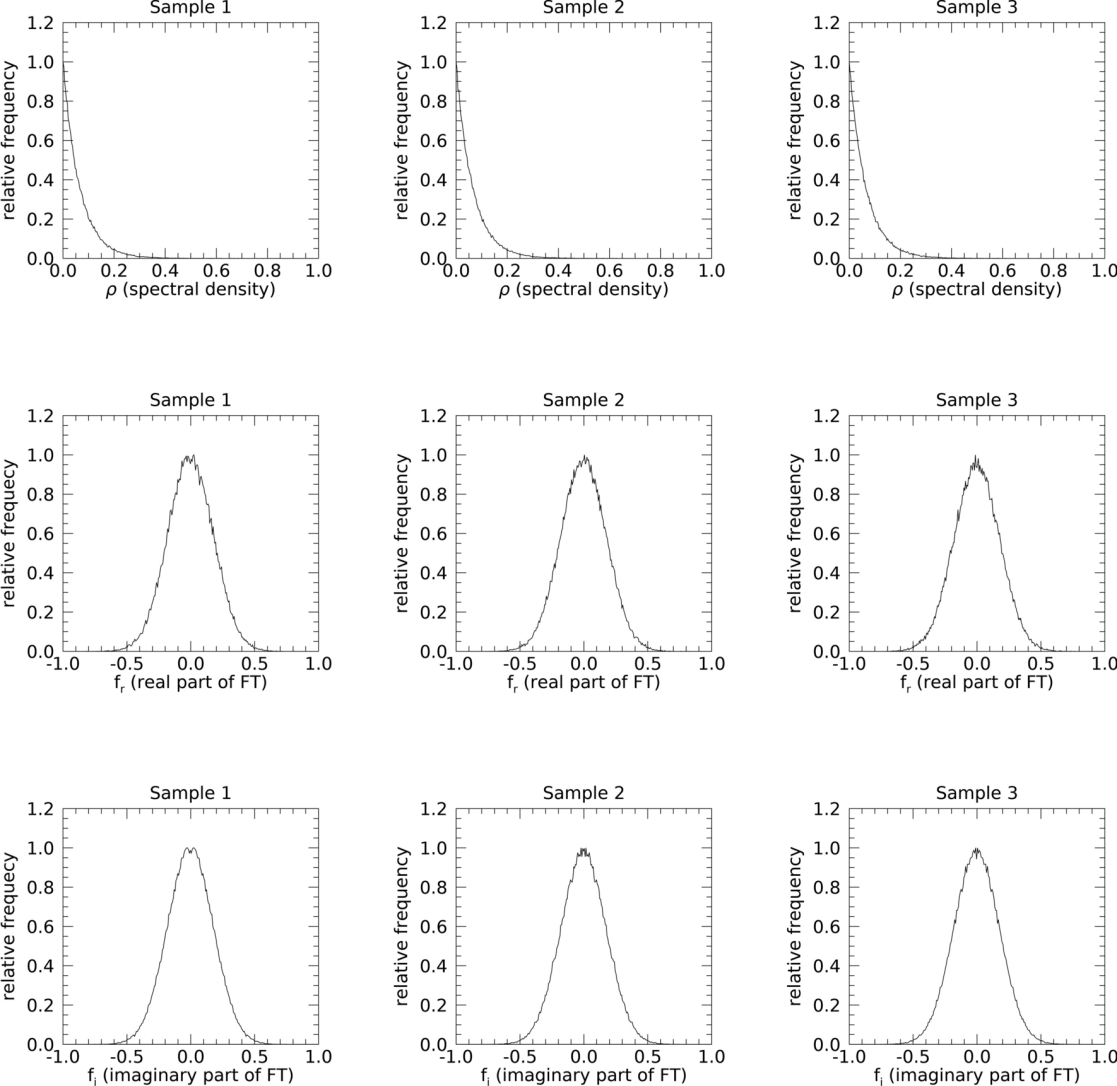
Fourier domain constituent empirical probability density function samples: the top row shows the power spectra empirical probability density function (pdf) approximations, [g_1_(ρ), g_2_(ρ), g_1_(ρ)], for the three samples shown in Fig. 9. The middle row shows the respective empirical pdfs for the real parts (f_r_) of their Fourier transforms (FTs) and the bottom row shows the respective pdfs for their imaginary parts (f_i_) of their FTs.

The real and imaginary parts of the FT were added quadratically for each sample image, and the empirical pdfs were first assessed visually. The respective pdfs for the FD images in Fig. 9 are shown in Fig. 10 with the same ordering. In the top row (left pane), the exponential form for g_1_(ρ) is expected when its components are distributed identically normal. The pdfs for SDN, [g_2_(ρ), g_3_(ρ)], have a similar exponential form (shown in the middle and left panes). The pdfs for the real and imaginary components for WN (left pane in the second and third rows) appear normal as expected. Note, the pdfs for the SDN components (middle and right panes in the second and third rows) also appear normal. In the ID, SDN images are not distributed normally but have pdfs that more resemble steeples with opposite concavity than the normal form (not shown). A plausible argument for normality in the FD and the spectra pdf-form may follow from the Central Limit Theorem. For a given SDN image, each point in the FD was derived by adding n^2^ (n = 500) random variables formed by the same operations in the construction of its real and imaginary parts.

**Fig. 11:**
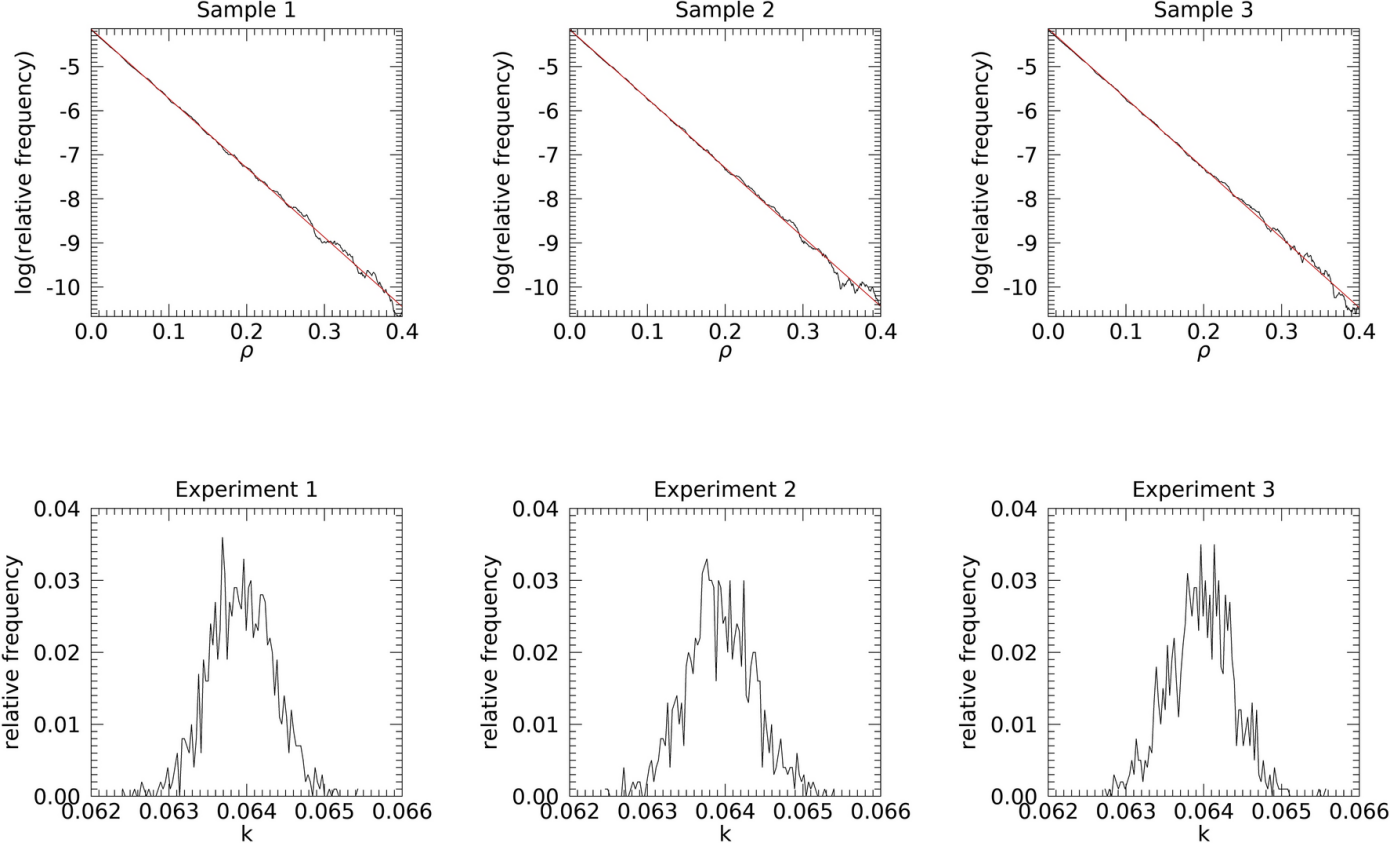
Power spectra probability density function modeling: the top plots show the natural logarithm of the probability density function (pdfs) from the power spectra (black) modeled as an exponential function and analyzed with linear regression analysis (red) for the samples from each experiment (top row, Fig. 10). The negative inverse of each slope gave: k_1_ ≈ 0.063, k_2_ ≈ 0.063, and k_3_ ≈ 0.064. The standard error (SE) ≈ 0.01 for each k_i_, and R ≈ − 1.0 for each plot. The bottom row shows the pdfs top for k_i_ derived from the 1000 samples from each experiment with distribution means: E[k_1_] ≈ 0.063; E[k_2_] ≈ 0.063; and E[k_3_] ≈ 0.063 with E[R] ≈ − 1.0. SEs were parasitic for each distribution mean. Summaries from the empirical k-distributions (bottom row) and correlation distribution reinforce the findings from the samples (top row).

The exponential parametric modeling of the power spectra was addressed with the regression analyses using Eq. (16). The linear k-plots for the samples are shown in the top row of Fig. 11. The negative of the inverse slopes, their SEs, and correlation coefficients were similar for these samples: E[k_1_] ≈ 0.063, E[k_2_] ≈ 0.063, and E[k_3_] ≈ 0.064 with E[R] ≈ − 1.0 and SE ≈ 0.001 for each E[k_i_]. These quantities are in close agreement with the power spectra distribution figures of merit. Likewise, the linearity indicates close agreement with the exponential model. The bottom row of Fig. 11, from left to right, shows h_1_(k), h_2_(k), and h_3_(k) derived from the 1000 samples from each experiment. Each experiment had similar distribution spatial means: E[k_i_]≈ 0.063 with parasitic SEs; and E[R] ≈ − 1.0. Similarly, these quantities are in close agreement with the theoretical values. Next, shorthand was used to refer to the experimental indexing for the statistical comparisons. The h_i_ (i = 1, 2, or 3) pdf pairwise comparisons with the KS test gave: p = 0.603, when comparing 1 with 2; p = 0.024 when comparing 1 with 3 (indicating a significant difference); and p = 0.304 when comparing 2 with 3. Comparing pairwise expected means, E[k_i_], from the h_i_ distributions with the t-test produced: p = 0.954 when comparing 1 with 2; p = 0.104 when comparing 1 with 3; and p = 0.129 when comparing 2 with 3. The KS test comparisons showed that experiments 1 and 3 deviated significantly and the t-tests indicated the means were not significantly different. Possible explanations for the KS test difference may be that the **F**[m(x, y)] filter kernels include anatomical structure represented in the FD not included in either **F**[g(x, y)] and **F**[n(x, y)], or it could be a statistical fluctuation artifact. Nevertheless, the close agreement with the exponential model provides indirect evidence that the real and imaginary components for SDN were well approximated as normally distributed.

**Fig. 12:**
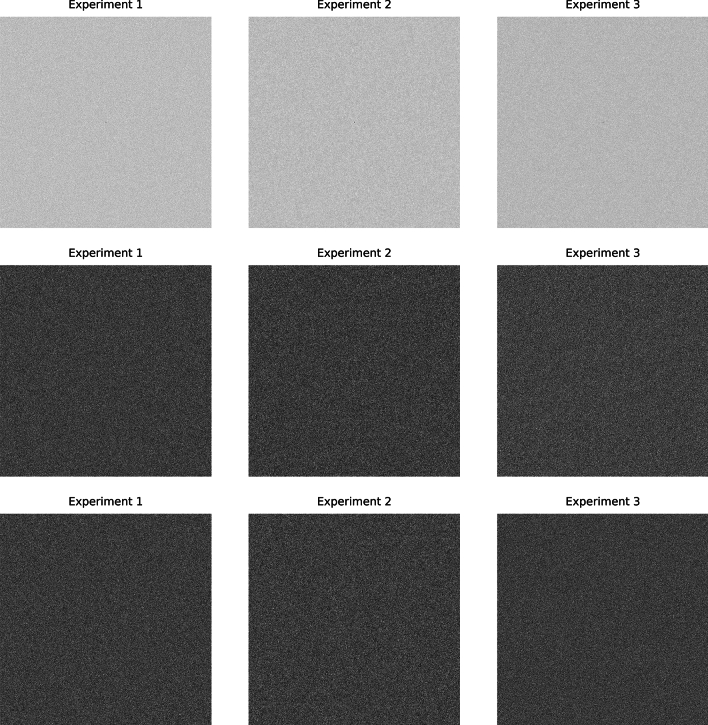
Fourier domain mean ensemble images: top row shows the ensemble mean power spectra images, ρ(avg), for each experiment. The real component mean-ensemble images, f_r_(avg), are shown in the middle row and the corresponding imaginary component mean-ensemble images, f_i_(avg), in the bottom row. Intra row wise comparisons appear similar as uniform chatter.

**Fig. 13:**
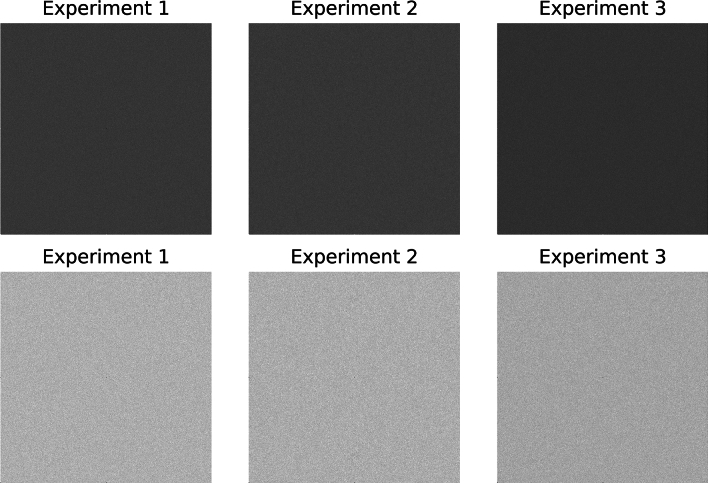
Fourier domain ensemble images of the variance for each experiment: top row shows the real component variance-ensemble images, f_r_(var), for each experiment, and the second row shows the corresponding imaginary component, variance-ensemble images, f_i_(var). Intra row wise comparisons appear similar and uniform.

**Fig. 14:**
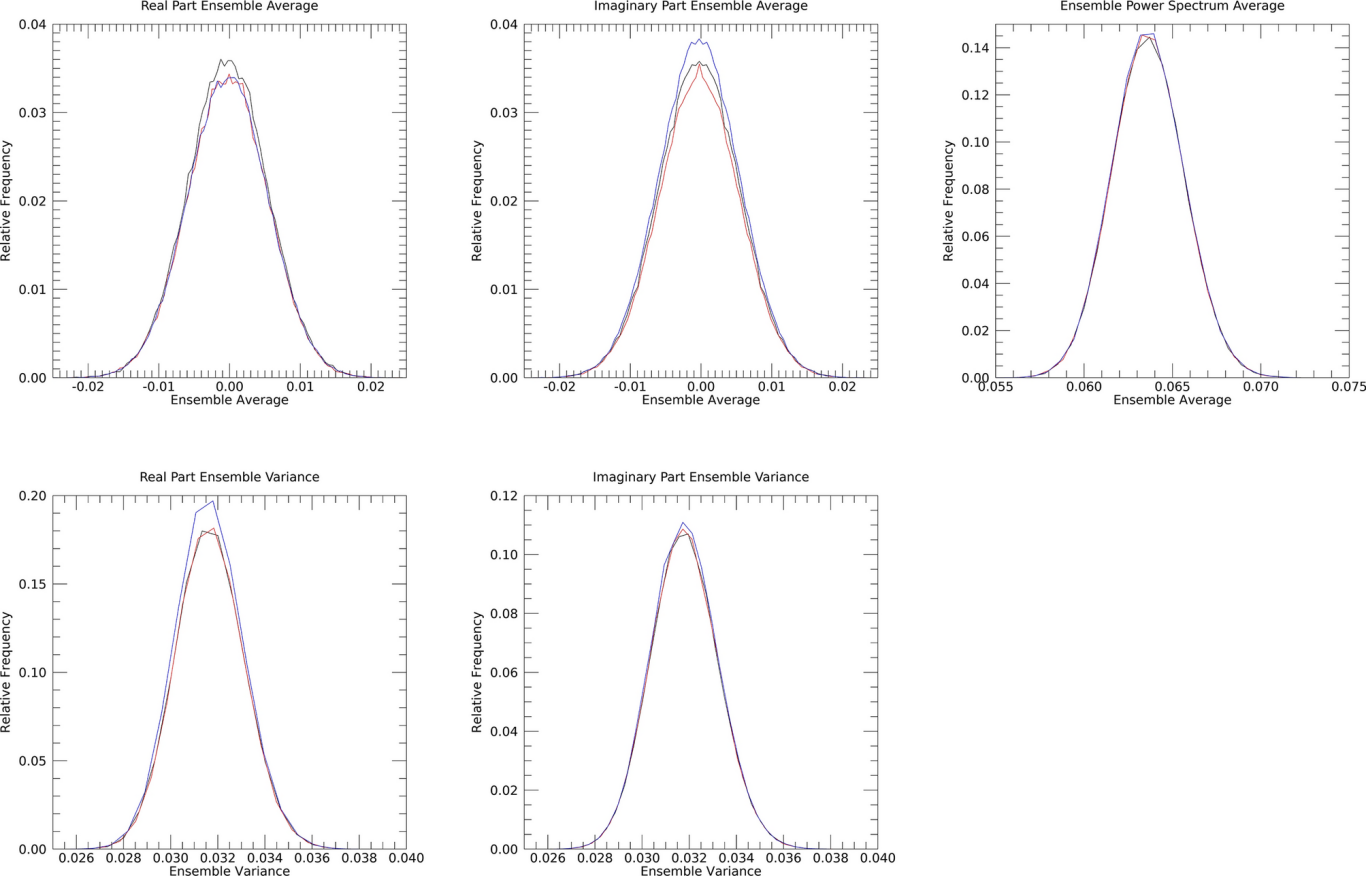
Ensemble Fourier domain empirical probability density function (pdf) comparisons for each experiment: in these plots, black, red, and blue curves correspond to experiments 1, 2, and 3 respectively. Each pane has the three curves derived from each experiment over-plotted. In the top row, the left pane shows the pdfs from f_r_(avg), the middle pane shows pdfs from f_i_(avg), and the right pane shows pdfs from ρ(avg). The second row shows pdfs from f_r_(var) in the left pane and pdfs from f_i_(var) in the right pane. Within each pane, the curves by observation show agreement.

For the ensemble findings, image illustrations and related summaries are discussed first followed by pdf comparisons. Figure 12 shows the ρ(avg) images in the top row. For each experiment, E[ρ(avg)] ≈ 0. 064 with parasitic SEs. Note, these findings are the expected values of k_i_ and agree with the exponential pdf modeling (i.e., spatial modeling) results. The f_r_(avg) images are shown in the second row, and for each experiment E[f_r_(avg)] ~ 0.0 with parasitic SEs. Likewise, the f_i_(avg) images are shown in the third row and for each experiment E[f_i_(avg)] ~ 0.0. As observable in Fig. 12, the images in each row appear uniform and similar. Figure 13 shows the expected value variance ensemble images sorted by row. The f_r_(var) images are shown in the top row and for each experiment E[f_r_(var)] ≈ 0.032 with parasitic SEs. Similarly, f_i_(var) images are shown in the bottom row and for each experiment E[f_i_(var)] ≈ 0.032 with parasitic SEs. As observable in Fig. 13, the ensemble expectation images in each row appear uniform across the Fourier plane and similar. Ensemble pdf comparisons are shown in Fig. 14. Here, black, red, and blue curves correspond to experiment 1, 2, and 3 respectively. The top row (left to right) shows the pdfs from f_r_(avg), f_i_(avg), and ρ(avg). The second row (left and right panes) show pdfs from f_r_(var) and f_i_(avg). There were insignificant differences between all pairs of pdfs when making inter-experiment comparisons with the KS test for a given Fourier constituent (1 with 2, 1 with 3, and 2 with 3); all gave p ~ 1.0 (testing all pdfs from rows one and two). Table 3 gives the related t-test findings derived from comparing the mean and variance distributions. Both the KS and t-tests resulted in insignificant differences between WN and SDN or between SDN noise types, agreeing with the Fourier plane spatial analyses. The similarity with WN by both observation and statistical test findings are indications that SDN is statistically uniform in the FD.

**Table 3:** Fourier domain t-test ensemble image distribution comparisons.

Fourier constituent	Comparisons 1 vs 2	Comparisons 1 vs 3	Comparisons 2 vs 3
1. Real component: f_r_(avg)	0.52	0.99	0.53
2. Imaginary component: f_i_(avg)	~ 1.0	~ 1.0	~ 1.0
3. Power spectrum: ρ(avg)	~ 1.0	~ 1.0	~ 1.0
4. Real component: f_r_(var)	0.68	0.89	0.56
5. Imaginary component: f_i_(var)	0.74	0.91	0.65

p-value approximations are cited for comparing inter-experiment distributions derived from the respective ensemble images: the expected mean distributions (rows 1–3) for the parenthetically referenced quantity and the expected ensemble variance distributions (rows 4 and 5). Experiments are referenced as 1, 2, or 3 in the top row. All comparisons within a row showed insignificant differences.

Because of the controlled ID conditioning (mean centering, variance normalization, and fixed dimensionality), it would be expected that simple averaging of the spatial findings and the ensemble averages would to be statistically similar (averaging order can be switched as the same pixels were included in the totals) in the FD for a given experiment or when making comparisons across experiments when the Central Limit Theorem applies. The statistical uniformity conclusion more strictly follows from the variance analyses of the real and imaginary components of the FTs: pdfs of the spatial area variances and ensemble variances had parasitic distribution variances; and SEs in their expected variance quantities were also parasitic. Because the number of elements used in these expectation calculations were relatively large (either 500 × 500 or 1000), we would expect the various summaries to approach their respective theoretical values. The same reasoning applies to the parametric modeling parameters when the models are correct. These arguments should be taken in the context that both the wavelet expansion and FT conserve the variance. Thus, the experimental variation was relatively small. These investigations demonstrated that switching the related ensemble statistics with the spatial statistics was approximately valid for SDN as well. In general, Strategy 4 resulted in showing close similarity between WN and SDN.

### Additional analyses

**Fig. 15:**
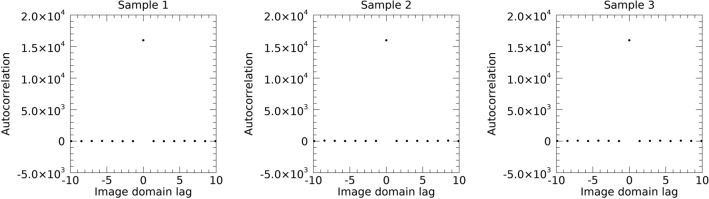
Inverse Fourier transform of the power spectrum for each sample: the inverse Fourier transform of the power spectrum for each sample produced a *delta function* in the image domain. This is an indication that the power spectra were all statistically uniform.

**Fig. 16:**
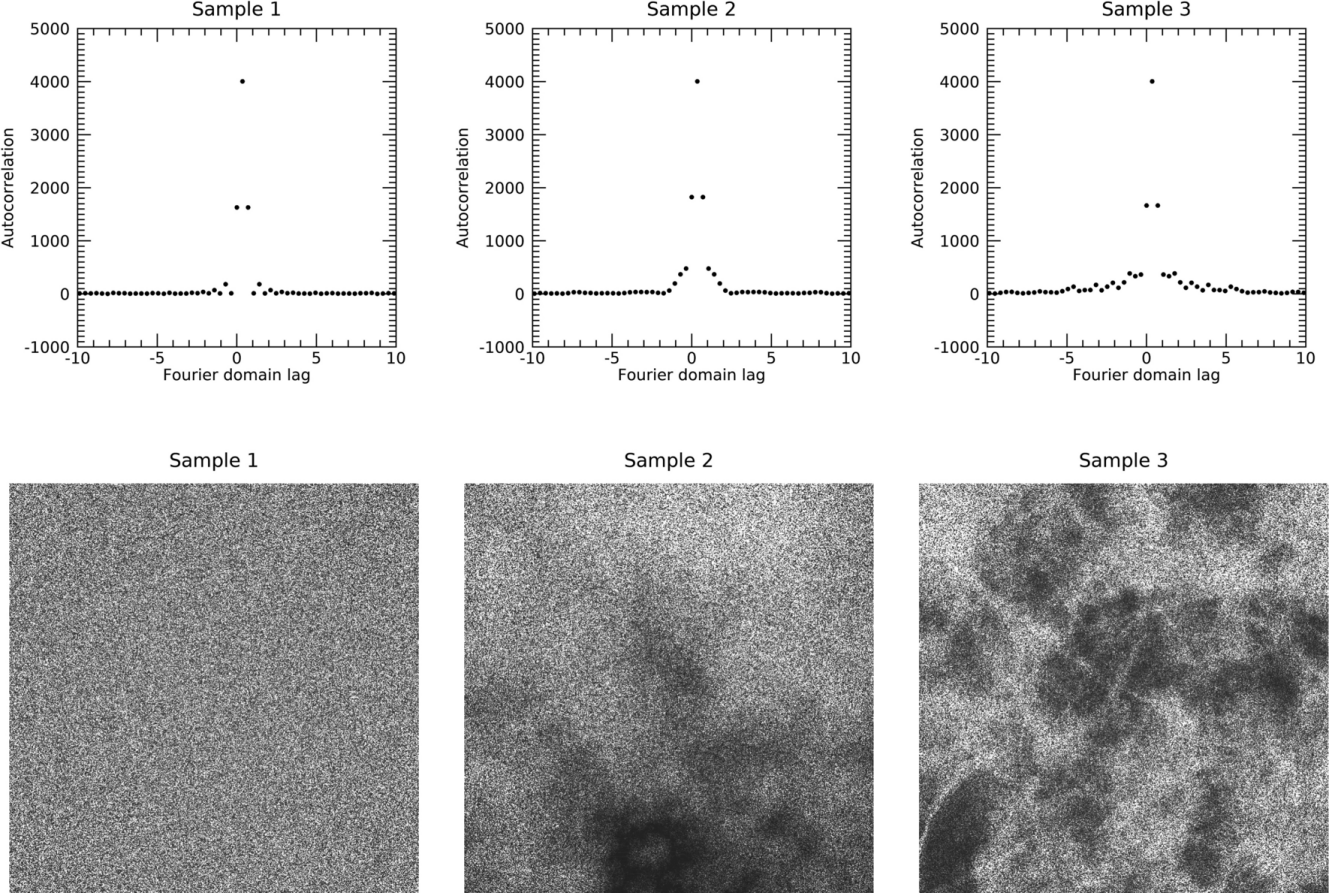
Fourier domain lag-autocorrelation function for each sample: the top row shows a line through the diagonal of the 2D lag-autocorrelation function (magnitude) of the Fourier transform for each sample. Sample 1 resulted in a *delta function*, whereas the other samples showed short range lag-correlation. The bottom row shows the square root magnitude of the inverse FT of the Fourier domain lag-autocorrelation function for each sample. Sample 1 is a uniform random noise image as expected, whereas samples 2 and 3 show close resemblance to their respective g(x, y) and m(x, y) images [see Figs. 5 and 6, respectively] as predicted by the work in the Appendix.

Findings from heretofore indicated SDN does not differ from WN, particularly in the FD, which was an unexpected overall result. It appears that there may be coherent signal embedded in the h_0_ images in experiments 2 and 3 (especially in Experiment 3) not fully accounted for directly in the FD with the planned analyses performed above. Therefore, two brief additional analyses were undertaken by studying the sample image from each experiment to detect possible differences in the lag-autocorrelation performed in the ID and FD. Additional analysis 1 addressed the ID spatial correlation by taking inverse FT of the power spectra. Each inversion resulted in a delta function at the origin as expected, also indicating similarity between WN and SDN. Figure 15 shows the diagonal lines through the respective ID lag-autocorrelation images, where all plots show a large spike at zero-lag. These *delta function* findings agree with the one-dimensional (1D) derivation provided in Appendix A1 and FT theory (i.e., for a given image, the power spectrum and lag-autocorrelation function are FT pairs, or more generally a flat or top-hat function in one domain is a *delta function* in the conjugate domain). The delta function approximation is expected when the power spectrum is uniform, as is the case for WN. This also indicates the spectra for SDN is uniform (agreeing with the FD analyses). Additional analysis 2 addressed correlation in the FD. This approach follows from the 1D derivation in Appendix A2. When extending this derivation to 2D, it predicts the lag-autocorrelation, of either **F**[s(x, y)] or **F**[n(x, y)] performed in the FD, defined as R(**w**), results in autocorrelation of the filter kernel for samples 2 and 3, where **w** is the complex 2D lag variable in the FD. This also applies to Sample 1 by letting n(x, y) = g(x, y) × n(x, y) with g(x, y) = 1. These findings are best interpreted as statistical estimations from random signals. It follows, the inverse FT, **F**^-1^[R(**w**)] should produce images that are ~ g(x, y)^2^ or m(x, y)^2^ for SDN and uniform (flat) chatter for n(x, y). A diagonal line through |R(**w**)| for each sample is shown in Fig. 16 (top row). Sample 1 produced a delta function (left pane) as expected because it was derived from complex random images. In contrast, the diagonals for samples 2 and 3 show short range lag-correlation in the FD (middle and right panes, respectively). It is interesting to note that the correlation functions for samples 2 and 3 differ. This would be expected because experiment 2 is a totally random process, whereas experiment 3 contains anatomical structure. The findings from samples 2 and 3 are indications that their FTs exhibit finer-scale texture due to the effective restricted spatial extent of their lag-autocorrelation functions. The bottom row shows the sqrt(|**F**^-1^[R(**w**)]|^2^) images for each sample. By observation, Sample 1 is a uniform noise image as expected, whereas samples 2 and 3 produced images that resemble their respective g(x, y) and m(x, y) shown in Figs. 5 and 6 as expected. The lag-autocorrelation was performed in the FD that produced images that were 2 × n − 1 pixels in each dimension. The inverse FT produced images with the same 2 × n-1 dimensions (giving interpolated pixels, not a larger spatial field of view). Images in the bottom row of Fig. 16 were reduced to their original n × n size with a cubic convolution procedure. The additional findings illustrated in Figs. 15 and 16 agree with the derivations in the Appendix. The findings from these three sample images show the only differences this study found in the FD between WN and SDN, and between the two types of SDN.

## Discussion

This study characterized WN, and its established (and known) attributes were used as reference for comparisons with SDN undergoing the same analyses. The general approach was to study how these images behaved under the wavelet and Fourier transforms and connecting the two transforms with FD illustrations. The inverse wavelet transform was described as an expansion in the ID or equivalently as summing the outputs from an orthogonal filter bank that decomposes the input. WN was used as the filter bank input, and the variances of the filtered output images (i.e., the decomposed variance) were parametrically modeled. This ID expansion was described in parallel with its FD spectral distribution illustrations. The theoretical equation predicts that the variance for WN decomposes exponentially as a decreasing function of spatial frequency band location, agreeing with the experimental findings; SDN followed the same exponential model and was distributed similarly (spatially) in the FD. As a given (theoretically) in the FD, WN has these attributes: identically normally distributed real and imaginary components; and a uniform power spectrum distributed exponentially. Both the observational and analytical findings for WN agreed with these expected theoretical attributes. In sum, SDN obeyed the same wavelet-based variance decomposition model and had the same Fourier attributes from both the spatial and ensemble analyses as WN except for the lag-autocorrelation findings in the FD. These FD correlation findings suggest there may be a way to characterize a given SDN image, or m(x, y) image, outside of the SDN environment. It follows, the long-range spatial correlation in the ID (known characteristic of mammograms or natural images) produced concentrated spatial attributes when the correlation was examined in the FD.

Similarities between WN and SDN are counterintuitive, as WN is spatially stationary in the ID, in contrast with SDN generally. Three complex random processes were created in the FD from the respective ID ensembles defined by the images in each experiment. As above, we considered the FD constituents, *equivalent* to images. SDN images were constructed by multiplication in the ID. For SDN, each complex point in the FD was created by the same two linear transformations (FT followed by a 2D convolution) operating on the same collection of ID points (i.e., **F**[n(x, y)] convolved with **F**[(s(x, y)]). It follows, each point in the FD is a complex random variable with the same expected mean and variance, producing uniformity across the Fourier plane as found in here. For Experiment 2, we conclude the related FD process is to a good approximation stationery. As, each **F**[g(x, y)] is statistically similar for each **F**[s(x, y)] realization; the image formation mechanism is static and not a function of t. It follows, the statistical properties of this process should be static over t. The same argument does not apply to Experiment 3 because m(x, y) differs for each s(x, y) realization, unless the collection of **F**[m(x, y)] are statistical realizations of the same random process. When this is the case, this process may also be stationary in the FD. Experiment 3 is a random function of t. Thus, its perturbation does not parallel a more controlled nonstationary process such as random thermal voltages changing in time because of a slow temperature climb for example, but it more resembles *chaotic* change (like Experiment 2). Perhaps the measurements analyzed in this study were too coarse to detect differences. Observations from Experiment 3 will require further investigations.

FD positive uniformity gives rise to a related Fourier pair relationship that can be exploited as well for describing WN-SDN similarities. Because n(x, y) is stationary, its lag-autocorrelation from a single observation is also its ensemble correlation function, which is incidental; the FT of its lag-autocorrelation function produces a uniform power spectrum. As demonstrated, the power spectrum for SDN was statistically uniform; the inverse FT produced the same ID lag-correlation as WN. By ID observation, WN obviously differs from SDN, suggesting there may be differences in the FD as found here in the lag-autocorrelation functions.

A more precise analysis of the practical importance of spatial correlation in the FD is warranted and is planned research. This finding may be a data reduction approach when attempting to describe a given image within or outside of the SDN context. More specifically, this approach could also be applied to a native mammogram or natural scene (without noise multiplication) because its FD characteristic is well concentrated about zero-lag. This is in contrast with the respective FT amplitude decay or ID characterization. The derivation shows that FD lag-autocorrelation function’s FT pair is the magnitude squared of the ID image before noise multiplication. The related derivation in the Appendix is more general than the findings here. When filtering identically distributed stationary random noise, the lag-autocorrelation is the filter’s lag-correlation, independent of the domain, agreeing with the development by Bracewell^73^ and findings from noise research in magnetic resonance^74^. One innovation in this study was applying this type of analysis in the FD.

Although this investigation characterized many aspects of SDN, there are some qualifiers that are best addressed. Relatively large ROIs (sometimes termed image patches) were investigated in this paper. We expect the wavelet expansion modeling findings will apply to full-size mammograms. Moreover, the related FD distribution illustrations will apply to regions that approximate the full mammogram as well, such as the largest rectangle that can fit within a given breast area^75^. The extension to smaller ROIs within a given image than considered here requires further study. For example, we would expect the variance decay measurements to show greater variability as the ROI is reduced to some critical size. Phase information in the FD was not investigated directly and is planned research. Although often neglected in favor of the Fourier magnitude, there are situations where the phase information is more important such as for image reconstruction^76^ and lesion visibility in mammography^77^. It is interesting to note, the analysis of natural scenes showed that phase data was predominate for image reconstruction when studying large patch sizes (i.e., studying image filling patches) relative to the image size, whereas the Fourier magnitude was predominant for relatively smaller patch sizes^78^. Further analyses are required to determine where our work fits within this patch-size framework. The findings in this paper were derived from simulations (either totally or in part derived from clinical mammograms) and represent idealized situations. However, the SDN archetype investigated here is used as a factor when modeling various image acquisition systems as discussed in sections "Signal dependent noise prevalence" and "Signal dependent noise modeling".

The purpose of this study was to investigate possible similarities between SDN and WN. However, the work has parallels with the objectives of other research and possible practical applications. For the most part in these related studies discussed in sections "Signal dependent noise prevalence" and "Signal dependent noise modeling", the SDN component was not studied in detail or in isolation in either domain with the methods used in this study. While our purpose was not noise reduction or image reconstruction, the power of the SDN term can be attenuated considerably with a linear filtering process because ¾ of its variance can be blocked with a (linear) high-pass half-band 2D filter, as demonstrated. When I(x, y) tends to lower spatial frequencies as in natural scenes, western art^79–81^, mammography, or medical images in general^82^, the findings found here may be useful for noise reduction purposes. The work also demonstrated that the image factor in the multiplicative noise model component could be recovered by applying the lag-correlation in the FD, but more work is required to apply this method to the entire SDN model. This study found that SDN could be parametrically modeled in the FD. We posit the possibility of multiplying a mammogram by noise and taking its FT as an analysis step. This produces random noise images with known parametric distributions as demonstrated. Multiresolution statistical tests could be developed for abnormal mammograms by considering the respective area in the FD where such abnormalities may have an elevated signature that skews the expected known distribution (normal distributions with known lag-autocorrelation functions for example). This supposition requires that the FD signature of an abnormality of interest survives the noise multiplication in the ID and is detectable in the FD, which requires further research to substantiate. We note, the analysis of the FD is not the choice for some authors when searching for metrics that scale^83^. However, the lag-correlation performed in the FD is both a novel analytical approach and finding, and it is not constrained to SDN studies. The limited extent of R(**w**) could prove useful for characterizing or developing similarity metrics for comparing images in the FD.

## Conclusion

In sum, this study investigated both WN and SDN by characterizing their attributes after undergoing commonly used transforms. The study showed the wavelet expansion-based variance decomposition of WN attenuates exponentially as does SDN. The study also showed that SDN has a uniform power spectrum that is distributed identically at each coordinate in the FD, agreeing with the theoretical expectations for WN. In the FD, SDN behaved as a stationary ensemble in some attributes; although to prove this, it will be necessary to show the lag and ensemble autocorrelation functions are statistically similar. The FD constituents of SDN require further analyses to describe their possible differences with WN that were not investigated in this paper. The lag-autocorrelation performed in the FD may be a method to characterize or compare images, which is planned research.

## Supplementary Information

Supplementary Information.

## Data Availability

Mammography data can be obtained upon request to the corresponding author with a data transfer agreement: John Heine (john.heine@moffitt.org).
