# Supplementary material for: Fourier analysis of signal dependent noise images
Source: Sci Rep. 2024 Dec 28;14:30686. doi: 10.1038/s41598-024-78299-1 (PMC11681241; doi:10.1038/s41598-024-78299-1)
Supplement: Supplementary file 1 — Supplementary Information. [file 41598_2024_78299_MOESM1_ESM.docx]

**Appendix**

A1*. Image domain lag-autocorrelation for signal dependent noise*

A one-dimensional (1D) derivation is provided to show why the lag-autocorrelation for signal dependent noise (SDN) should approximate a delta function. The idealized zero mean SDN signal in the reception domain (i.e., a 1D signal) can be expressed as the elements of a transposed column vector with real elements given by

| $z_{i}^{T}={[a}_{1}\times n_{1}, a_{2}\times n_{2}, \ldots,a_{i}\times n_{i},\ldots,a_{\infty}\times n_{\infty}]$ | Eq. (A1) |
| --- | --- |

for integer i > 0, where a_i_ are uncorrupted signal values not all zero or equal, n_i_ are zero-mean identically distributed stationary noise realizations, the superscript T defines transpose, and the index, i, is the discrete independent variable increment or position. We are considering a signal that is long in spatial extent and will examine lag in one direction as the lag autocorrelation function is symmetric about zero lag in 1D. A shifted version of z is given by the

| $z_{j}^{T}{=[a}_{1+j}\times n_{1+j},{a_{2+j}\times n}_{2+j} , \ldots, a_{i+j}\times n_{i+j},\ldots,a_{\infty}\times n_{\infty}]$ | Eq. (A2) |
| --- | --- |

for j > 0. The expectation of the inner product between z and shifted z produces the j^th^ shifted point in the lag-autocorrelation function given by

| $\frac{1}{n_{\infty}}\times z_{i}^{T}z_{j} \approx0$. | Eq. (A3) |
| --- | --- |

This becomes evident with an equivalent expression

| $\frac{1}{n_{\infty}}\sum_{i} {(a}_{i}\times a_{i+j})\times(n_{i}\times n_{i+j}) \approx0$, | Eq. (A4) |
| --- | --- |

which holds for j > 0. Because n_i_ are independent, it is reasonable to assume the expected value of the scaled noise factors to approximately cancel when n is large (i.e., $n_{\infty}$). When j = 0, the summation produces the variance, which is relatively large in comparison to the expectation when j ≠ 0. The same principle applies in 2D. Here, we have glossed over differences between the theoretical and discrete Fourier correlation relationships. Nevertheless, these principles are reasonable approximations.

A2*. Lag-autocorrelation in the Fourier domain*

A one-dimensional (1D) development is provided to detect possible differences between white noise (WN) and signal dependent noise (SDN) in the Fourier domain (FD), briefly addressed in the main paper. We have renamed variables used in the main investigations. Operations often *reserved* for the image domain (ID) are applied in the FD. The development starts with taking the Fourier transform (FT) of the 1D analog of Eq.(5a) or Eq. (5b). Rewriting Eq. (5) with relabeling gives s(t) = g(t)×n(t), where t is an arbitrary 1D reception domain variable, g(t) is a general function, and n(t) is stationary zero-mean random noise. Taking the FT of the modified 1D version of Eq. (5), using f as the conjugate variable, and applying the Fourier convolution theorem produces

| $S\left( f \right) = G\left( f \right)*N\left( f \right)=G*N$, | Eq. (A5) |
| --- | --- |

where capitals indicate the FD. Using abbreviated notation, the lag-autocorrelation of S(f) defined, as R(f), is given by

| $R(f) = (G*N)\star(G*N)$, | Eq. (A6) |
| --- | --- |

where the star symbol is the lag-correlation operator. Taking the inverse FT of Eq. (A6), applying the convolution and correlation theorems give

| $r(t)=(gn)(gn)^{\dagger}$, | Eq. (A7) |
| --- | --- |

where the dagger symbol indicates complex conjugate. Rearranging terms gives an equivalent expression

| $r(t) = (gg^{\dagger})(nn^{\dagger})$, | Eq. (A8) |
| --- | --- |

where r(t) is the magnitude squared of s(t). Taking the FT in the reverse direction and applying the Fourier correlation and convolution theorems again give

| $R(f) = (G\star G)*(N\star N)$. | Eq. (A9) |
| --- | --- |

The lag-autocorrelation for N(f) is approximately proportional to a delta function for long n(t) records

| $\int N(v)N^{\dagger}(v-f)dv\approx k\times\sigma(f)$, | Eq. (A10) |
| --- | --- |

where k is the variance of the n(t), and σ(f) is the delta function. The lag-autocorrelation for G(f) is given by

| $A\left( f \right)=\int G(\tau)G^{\dagger}(\tau-f)d\tau$. | Eq. (A11) |
| --- | --- |

To determine R(f) in Eq. (A9), we use the definition of the convolution integral with Eq. (A11), reverse the delta function and shift it by w giving

| $R\left( w \right)= k\times\int A\left( f \right)\sigma\left( w-f \right)df=k \times A(w)$, | Eq. (A12) |
| --- | --- |

where w is a dummy spatial frequency shift variable in the FD. This shows that lag-autocorrelation performed in the FD is the autocorrelation of the filter kernel in Eq. (A11) within a scale factor, differing from FD lag-autocorrelation function for WN in Eq. (A10). The inverse FT of Eq. (A12) gives ~ g^2^(t).
